# Controlling Working Memory Operations by Selective Gating: The Roles of Oscillations and Synchrony

**DOI:** 10.5709/acp-0199-x

**Published:** 2016-12-31

**Authors:** Mario Dipoppa, Marcin Szwed, Boris S. Gutkin

**Affiliations:** 1 Institute of Neurology, Faculty of Brain Sciences, University College London, UK; 2 Group for Neural Theory, LNC U960 IEC , Ecole Normale Superieure PSL * University.; 3 Université Pierre et Marie Curie, Paris, France; 4 Departement of Psychology, Jagiellonian University, Kraków, Poland; 5 Center for Cognition and Decision Making, NR U HSE , Moscow, Russia

**Keywords:** working memory, neural oscillations, neural networks, selective gating, persistent activity

## Abstract

Working memory (WM) is a primary cognitive function that corresponds to the ability to update, stably maintain, and manipulate short-term memory (ST M) rapidly to perform ongoing cognitive tasks. A prevalent neural substrate of WM coding is *persistent neural activity*, the property of neurons to remain active after having been activated by a transient sensory stimulus. This persistent activity allows for online maintenance of memory as well as its active manipulation necessary for task performance. WM is tightly capacity limited. Therefore, selective gating of sensory and internally generated information is crucial for WM function. While the exact neural substrate of selective gating remains unclear, increasing evidence suggests that it might be controlled by modulating ongoing oscillatory brain activity. Here, we review experiments and models that linked selective gating, persistent activity, and brain oscillations, putting them in the more general mechanistic context of WM. We do so by defining several operations necessary for successful WM function and then discussing how such operations may be carried out by mechanisms suggested by computational models. We specifically show how oscillatory mechanisms may provide a rapid and flexible active gating mechanism for WM operations.

## Introduction

 We use working memory (WM) in our daily lives to perform a multiplicity of high-level cognitive tasks, like planning, speaking, reasoning, language comprehension, and thinking (Baddeley, 1992; Funahashi, 2006). To successfully support these cognitive tasks, WM needs to perform the following operations: temporarily store short-term memory (STM) information, flexibly manipulate this information, and be shielded from external distractors. While the brain circuit mechanisms that allow active maintenance of the WM trace has been largely identified, the neural mechanisms implementing other WM operations remain a matter of debate. Specifically, recent debate in the literature has centered around the mechanisms of selective gating that is necessary to perform the operations - or how does the WM system select relevant information to work with while ignoring the onslaught of irrelevant stimuli and errant thoughts. 

*Persistent activity*, the ability of neurons to remain in a high firing state after being activated by a transient stimulus, is thought to be playing a central role in WM. This persistent activity has been proposed to correspond to the memory buffer (Fuster & Alexander, 1971; Goldman-Rakic, 1995; Kubota & Niki, 1971; Riley & Constantinidis, 2015), to the encoding of abstract rules (D’Esposito & Postle, 2015) and control signals (Cohen et al., 1997) of WM, and to be supported by the local recurrent synaptic interactions (Goldman-Rakic, 1995). The activation and deactivation of persistent activity could thus be taken as the neural substrate of loading and clearing STM memories (or abstract rules). A key question is then, how are these WM dynamics controlled in order to execute the associated operations? 

 A general feature of the brain is the ubiquitous presence of pseudo-periodic neural activity, known as *neural oscillations*. For instance, execution of WM tasks is generally accompanied by either an increase or a decrease in power at different frequencies of neural oscillations in humans (Tallon-Baudry, Bertrand, Peronnet, & Pernier, 1998) and monkeys (Pesaran, Pezaris, Sahani, Mitra, & Andersen, 2002). Induced increases in oscillatory power during WM retention have been detected in the theta (4-8 Hz), beta (13-30 Hz), and gamma (30-200 Hz) ranges (Gevins, Smith, McEvoy, & Yu, 1997; Tallon-Baudry et al., 1998; Tallon-Baudry, Kreiter, & Bertrand, 1999), while the alpha range (8-13 Hz) has an active role in inhibiting information irrelevant for the WM task (Klimesch, Doppelmayr, Schwaiger, Auinger, & Winkler, 1999; but see Palva & Palva, 2007). Intriguingly, these oscillations follow an orderly sequence of specific frequencies progressing as the task unfolds from the start of the first stimulus to the behavioural response. Despite the prominence of oscillations during WM execution, their functional role is debated. 

 Computational models have proposed that the WM network could support mixing between low-frequency and high-frequency oscillations (Kopell, Whittington, & Kramer, 2011; Lisman & Idiart, 1995). Yet, these models struggled to explain why there is a progression of frequency content during WM tasks (Wimmer, Ramon, Pasternak, & Compte, 2016) and how the different bands are specifically related to the various necessary computational operations. At the end of this review, we will discuss how the modulation of these different oscillations has been exploited in an alternative model to allow the execution of a WM task by controlling the dynamics of persistent activity and hence WM operations (Dipoppa & Gutkin, 2013b). 

This review is structured in five sections. In section 2, we provide a review of WM with a specific focus on operations needed for the WM. We included a review of mapping WM in the brain and on the dynamics of persistent activity during WM tasks. In section 3, we present a focused review of the evidence for an active role of oscillations from the neuroscience literature on WM. In section 4, we present models that explain how the necessary operations we have defined may be implemented, with particular focus on gating models based on inhibition. We then show how a recent gating model based on neural oscillations (as opposed to inhibition) may resolve open questions with respect to the operations and the observed dynamics of the oscillatory activity. In section 5, we provide conclusive remarks and list main open questions concerning WM.

## Working Memory: Characteristics and Circuit Mechanisms.

 WM corresponds to the ability to memorize information for a limited period and actively use this retained information to perform cognitive and/or motor tasks ( Baddeley & Hitch, 1974). In other words, WM flexibly and actively manipulates, updates, and processes temporary memory traces (Cowan, 2008). The central characteristic of WM is that the active storage and the directed use of the information are inexorably and intimately linked. 

 Standard models of the WM system in the cognitive psychology literature consist of separate STM components and control signals, even though it is debated whether these STM components correspond to a generic activation of long-term memories present in the brain (Cowan, 2008) or correspond to areas specialized in a specific function (e.g., the phonological loop; Baddeley & Hitch, 1974). While the WM system as a whole could involve a distributed brain network (e.g., because of the activation of STM in specialized areas), the prefrontal cortex (PFC) is thought to play a central role in controlling the commands required to execute a delayed response task (Koechlin & Hyafil, 2007; Koechlin, Ody, & Kouneiher , 2003). Notably, the issue whether control signals are intrinsic to the WM as such or come from a more generic cognitive system, such as selective attention, are far from being settled. 

 In this review, we will pursue an interpretation aligned with proposals in systems neuroscience (Brunel & Wang, 2001; Machens, Romo, & Brody, 2005) that view the WM operations as integral within the neural circuits that maintain temporarily in memory the information required for the WM task. Central to this view is the encoding and maintenance of WM through persistent neural activity (Goldman-Rakic, 1995) and/or by dynamic encoding (Stokes, 2015). Since in the literature the term STM has assumed multiple meanings (most frequently it refers only to the short-term storage of information not including the manipulation of such information), in this review we will refer to working memory active storage (WMAS). We define WMAS in line with Cowan (2008) as temporarily activated long-term memory. The WMAS can be seen as the short-term storage component that is manipulated within the WM system and widely modelled with dynamical models (e.g., Amit & Brunel, 1997; Compte, Brunel, Goldman-Rakic, & Wang, 2000). 

 An important constraint is imposed on WM by its the severe capacity limitations ( Cowan, 2001; Luck & Vogel, 1997; G. A. Miller, 1956). The exact nature of this limitation is controversial: It has been proposed to correspond to a hard threshold on the number of items available in WM (Cowan, 2001; Luck & Vogel, 1997), or a non-discrete amount of resources that is shared between items (Bays & Husain, 2008; Ma, Husain, & Bays, 2014; Wilken & Ma, 2004), or a mixture of these models (Van den Bergh, Zhang, Arckens, & Chino, 2010; Zhang & Luck, 2008). In all of these accounts, the apparent number of distinct items that can be retained does not exceed a handful (between four and eight). Since the capacity is limited to only a few items, not only does WM need to store and maintain relevant information during the necessary time interval, but also to selectively gate the access to information within the WMAS (Frank, Loughry, & O’Reilly, 2001). This is in fact suggested by the rapid suppression of the memory-related persistent activity (Funahashi, Bruce, & Goldman-Rakic, 1989; Romo, Brody, Hernandez, & Lemus, 1999) and stability of these memory signals against interference by distracting stimuli (E. K. Miller, Erickson, & Desimone, 1996). 

 Attentional processes have also been functionally associated with WM (Cowan, 1995). For example, several lines of evidence suggest that attention plays a key role in the allocation of WM resources (Bays & Husain, 2008; Melcher & Piazza, 2011; Shao et al., 2010). Furthermore, it has been suggested that the nature of internal attention (directed to information held in STM) and external attention (directed to sensory information) are similar (Kuo, Rao, Lepsien, & Nobre, 2009; Nobre et al., 2004), with some subtle differences perhaps depending on how the features of the information (e.g., location and space) are processed (Van der Lubbe, Bundt, & Abrahamse, 2014). Yet, the circuit-based explanation of how selective gating is implemented, possibly by attention-dependent mechanisms, remains to be determined. 

### Working Memory Operations

**Figure 1.**
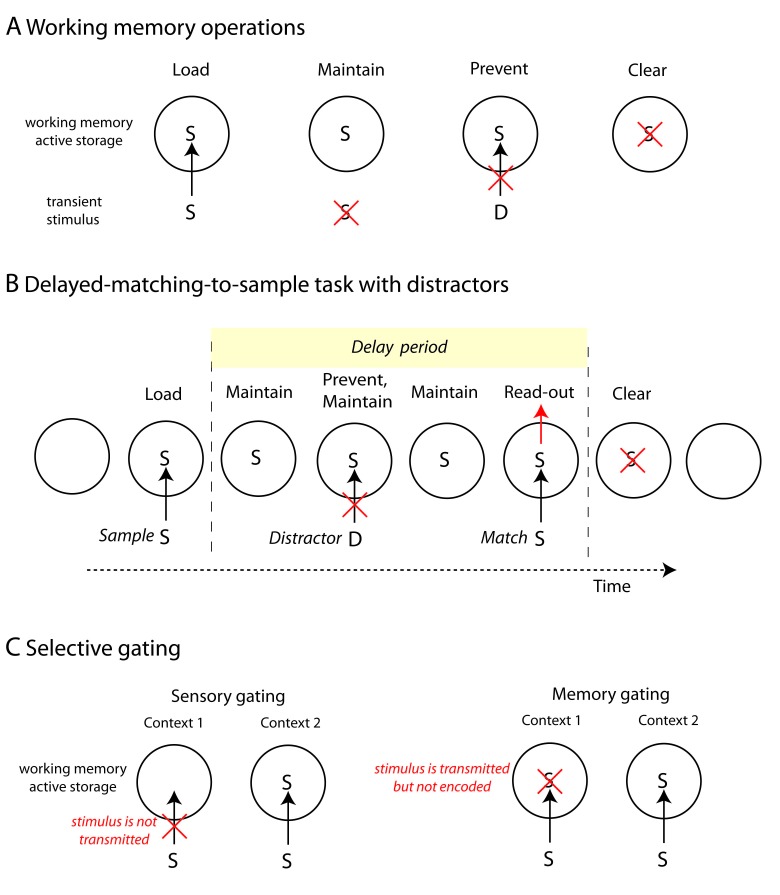
A) Working memory operations. Load: a stimulus (sensory or internally generated) is transmitted and encoded in the working memory active storage (WMAS). Maintain: after stimulus disappearance the stimulus is still kept in memory. Prevent: a distracting stimulus is prevented from disrupting the maintenance of a previously encoded stimulus (different mechanisms are laid out in C). Clear: an information previously encoded in the WMAS is actively cleared. B) WM operations during a delayed-matching-to-sample task with distractors: Load is performed at sample presentation. During the delay period the sample information is maintained and any distractor is prevented from disrupting such memory until read-out. After read-out the memory is cleared from the WMAS (alternatively the memory could passively fade-out in time). C) Two alternative selective gating mechanisms that, depending on the context, allow to prevent a stimulus from being encoded in the WMAS. Context 1: The stimulus is irrelevant and needs to be prevented. Context 2: The stimulus is relevant. If the mechanism is sensory-gating then the irrelevant stimulus is blocked before accessing the WMAS. In the memory gating mechanism the stimulus is transmitted INTO INTO the WMAS eliciting a transient response, but then it is not maintained in memory after stimulus removal.

Since the WM execution requires a sequence of cognitive operations, it is necessary to define them clearly. There are three basic operations that are commonly thought to be necessary to WM processing: *load*, *maintain*, and *read-out* (Machens et al., 2005; see Figure 1b):

• The load operation corresponds to encoding an item into the WMAS. The item could be afferent sensory information or internally generated. The important point is that this information must be necessary for task execution.

• The maintain operation corresponds to the successful retention of a memory in the absence of the stimulus during the required delay until the memory is used in task performance.

• The read-out operation refers to the capacity to use the relevant information held in memory in order to generate an appropriate response.

 In addition to these three core WM operations, the WM system also needs additional operations to overcome its capacity limitations. Notably necessary is the ability of WM to block distracting stimuli that could perturb the retention of relevant information (Hasher, Zacks, & May, 1999) in two ways: to *prevent* a distractor from entering the focus of attention (Hasher et al., 1999) or to *restrain* a prepotent stimulus to access WM and impede it from perturbing a previously encoded trace (Brunel & Wang, 2001; Hasher et al., 1999; see Figure 1a). Since both require a control over the access to the WMAS, we consider these as related processes and will refer to a unified prevent/restrain operation. 

 It is debated whether the removal of obsolete memories from the WM is caused by passive fading (Barrouillet, Portrat, Vergauwe, Diependaele, & Camos, 2011), interference from de novo appearing items (e.g., distractors; Oberauer & Lewandowsky, 2014), or by an active removal process (Brunel & Wang, 2001; Gutkin, Laing, Colby, Chow, & Ermentrout, 2001; Oberauer, 2001). We believe that the theory of passive memory decay is inconsistent with the finding that the recall performance of items is independent of the manipulation of delay intervals between the item presentations (Oberauer & Lewandowsky, 2014). A number of experiments suggest that at the end of a WM task, once the response is initiated, memories that are no longer useful are actively erased (Funahashi et al., 1989; Romo et al., 1999; see Figure 1a). 

 The operation of an active deletion of an obsolete memory is defined as *clear*. There are some suggestions that clear and read-out operations could be a result of a single process (Dipoppa & Gutkin, 2013b; Gutkin et al., 2001). 

 These three additional operations, restrain, prevent, and clear, are required by WM for proper function. However, it is still debated whether they are part of a more general attentional system in the framework of inhibitory control (Hasher et al., 1999) or if, as we advance in this review, they are intrinsic processes of the WM system (Cohen et al., 1997). The level at which an information is gated (transmitted or blocked) in the memory is a fundamental component of understanding the WM system. The operation of actively choosing, by whatever mechanism endogenous to the WM system or exogenous to it, is referred to as *gating*. In a heuristic sense it is a gate to the WMAS that is actively and selectively opened or closed, depending on task demands at different points in the task. As we will describe later, in section 3, memory can be gated via local inhibitory mechanisms (Brunel & Wang, 2001) or by modulation of endogenous neural oscillation (Dipoppa & Gutkin, 2013b). 

 One of the most common ways to measure the successful (or not) execution of the operations required by WM (in both humans and a selection of nonhuman species) is to test the subjects with delayed-response tasks. These delayed-response tasks possess a delay period, during which information should be retained, and a response period, during which the actively stored information should be used to execute an appropriate action. There is a large variety of delayed-response tasks that allow to measure different abilities of the WM system, such as the robustness of memory retention upon distractor presentation (e.g., in a delayed match-to-sample [DMS] task with distractors, E. K. Miller, Li, & Desimone, 1991; see Figure 1b) or such as the precision in WM (e.g., with tasks where a continuous variable is tested on an analogue scale, Wilken & Ma, 2004). 

 Let us now clearly delineate how the different computational operations we defined above map onto the structure of the delayed-response tasks. Within the context of the delayed-response tasks, the load operation corresponds to encoding an appropriate transient sensory stimulus (the sample or item) before the delay period (see Figure 1b). This stimulus can be a discrete object, as in the DMS tasks, or a parametric value of the item location as in the oculomotor delayed-response (ODR) task (Hikosaka & Wurtz, 1983). The maintain operation corresponds to the successful retention of the memory during the required delay period. The read-out operation refers to the capacity of using the relevant information to generate the correct response at the appropriate task phase. The operation clear is the ability of the system to erase a memory rapidly after the response phase, leaving the WM clear for subsequent engagement. The operation of prevent/restrain is the ability to block distractor stimuli that could access the working memory’s active storage (or the focus of attention) and perturb the retention of relevant information. While most theoretical models have primarily focused on these three operations, load, maintain, and read out, the operations prevent/restrain and clear have been often overlooked. In this review, we will specifically describe recent models that shed new light on possible mechanisms underlying these two last operations.

### Signal Gating versus Memory Gating

In fact, the process needed to perform the operations successfully is selective gating of information and access to WM. Interestingly, the gating could take two different forms: preventing the stimulus from reaching the WMAS at all (signal gating) or preventing the WMAS from converting the transient response caused directly by the sensory input into an active memory trace (memory gating).

Above we discussed WM frameworks where the distractors are transmitted to the working memory active storage (WMAS), leading to a transient response in the WM circuitry, but then intrinsic mechanisms of WM prevent them from (1) initiating a new erroneous memory trace; and (2) deactivating a previously activated persistent state or, more in general, a memory (memory gating, see Figure 1c). However, whether this scenario is valid for all types of WM remains an intriguing open question. In fact, distracting stimuli can also be blocked before they access the WMAS by top-down modulation or by local mechanisms (signal gating, see Figure 1c); in this case no, or a drastically reduced, transient stimulus-driven response should be seen in the WM circuitry.

 Signal gating can be executed in different ways. Some relevant examples include *amplitude signal gating* obtained by controlling the balance between excitation and inhibition in the receiver network (e.g., Vogels & Abbott, 2009) and *temporal signal gating* obtained by controlling the timing of excitatory and inhibitory signals (e.g., Fries, 2005; Kremkow, Aertsen, & Kumar, 2010). Again, the clear signature of such gating is a blockade of the transient response in the PFC neural activity. However, PFC neurons featuring selective persistent activity have a significant transient increase of activity at distractor presentation (E. K. Miller et al., 1996) that is at times even stronger than the corresponding response to the item. This effect suggests that even the irrelevant stimulus is able to reach the WMAS, and, therefore, the brain is using in this context a memory gating mechanism rather than signal gating. 

 It remains unclear if the gating control (such as gating relevant vs. irrelevant information) is part of the intrinsic processes of the WM or stems from a general cognitive system, such as selective attention, that is external to WM. Notably, computational models have demonstrated that the switch between different gating modes (from a memory mode where new stimuli are gated-in to a decision mode where stimuli are compared) can be obtained directly within the WMAS, without the need of external control modules (Chow, Romo, & Brody, 2009; Machens et al., 2005). Before we can focus on the gating modes, we need to map the WM system in the brain in order to be clear what brain circuits are involved. 

### Mapping the Working Memory System in the Brain

 Before we go on to argue how the selective gating mechanisms are implemented in the neural circuits that underlie the WMAS, let us review the functional anatomical organisation of the WM system. Performance in WM tasks is associated with a distributed activation of several brain areas (Baddeley & Hitch, 1974). This distributed system is consistent with the requirement for WM to retrieve different associated memories that are located in different specialized areas (Fuster, 1997) and different hemispheres (Funahashi, Bruce, & Goldman-Rakic, 1993). The PFC, an area that is thought to execute cognitive processes along with motor preparation and initiation, thinking and speech (Fuster, 1997), plays a central role in WM execution as demonstrated by lesion (Funahashi, Bruce, et al., 1993) and recording (Fuster & Alexander, 1971; Kubota & Niki, 1971) studies. In particular, in the monkey PFC, a population of neurons increases their activity level with respect to the baseline during the delay period subsequent to the stimulus offset (Fuster & Alexander, 1971; Kubota & Niki, 1971) in a manner that is stimulus identity selective (Fuster & Jervey, 1981). This effect was defined as persistent activity, and it was hypothesized that this neural state is associated to memory retention (Goldman-Rakic, 1995; Riley & Constantinidis, 2015; Wimmer, Nykamp, Constantinidis, & Compte, 2014). 

 It has been also proposed that persistent activity reflects high-level task contingencies and rules rather than storage of sensory stimuli (D’Esposito & Postle, 2015). In this framework, the PFC still plays a central role. For example, both monkey ( Rigotti et al., 2013; Warden & Miller, 2010) and human (S. H. Lee, Kravitz, & Baker, 2013; Riggall & Postle, 2012) studies show that PFC encodes more task rules and abstract representations than stimuli to be remembered. In monkeys, the dorsolateral PFC has been shown to encode spatial WM retention (Funahashi et al., 1989) as opposed to the ventrolateral PFC that encodes object WM retention (Fuster, Bauer, & Jervey, 1982). A specialization of WM retention has been proposed for humans as well: Spatial WM, for example, is encoded in the superior frontal sulcus as opposed to the object WM being encoded, as in monkeys, in the ventrolateral PFC (Courtney, Petit, Maisog, Ungerleider, & Haxby, 1998; Courtney, Ungerleider, Keil, & Haxby, 1996). 

 Outside the PFC, impairment or stimulation of specific areas has shown an involvement of different cortical areas, such as the temporal cortex for visuo-object information (Fuster, Bauer, & Jervey, 1981; Oliveri et al., 2001), the PPC for visuo-spatial information (Oliveri et al., 2001), and somatosensory cortex for haptic information (Harris, Miniussi, Harris, & Diamond, 2002). Furthermore, persistent activity during delay-response tasks has also been found to encode visuo-object information in the inferotemporal cortex (ITC; Fuster & Jervey, 1981; E. K. Miller et al., 1996), visuo-spatial information in the PPC (Constantinidis & Steinmetz, 1996), and haptic information in the somatosensory cortex (Zhou & Fuster, 1996). In the primary sensory areas, a delay period activity is often recorded in the form of a sub-threshold pattern of activity (i.e., not persistent activity, such as in the visual cortex; Serences, Ester, Vogel, & Awh, 2009). Several subcortical areas appear also to be part of the WM system as, for example, the basal ganglia, an area involved in selective disinhibition of the frontal cortex (Frank et al., 2001), and the midbrain with its dopaminergic nuclei, such as the ventral tegmental area.

### Persistent Activity in the Prefrontal Cortex as the Neural Basis of Working Memory

**Figure 2.**
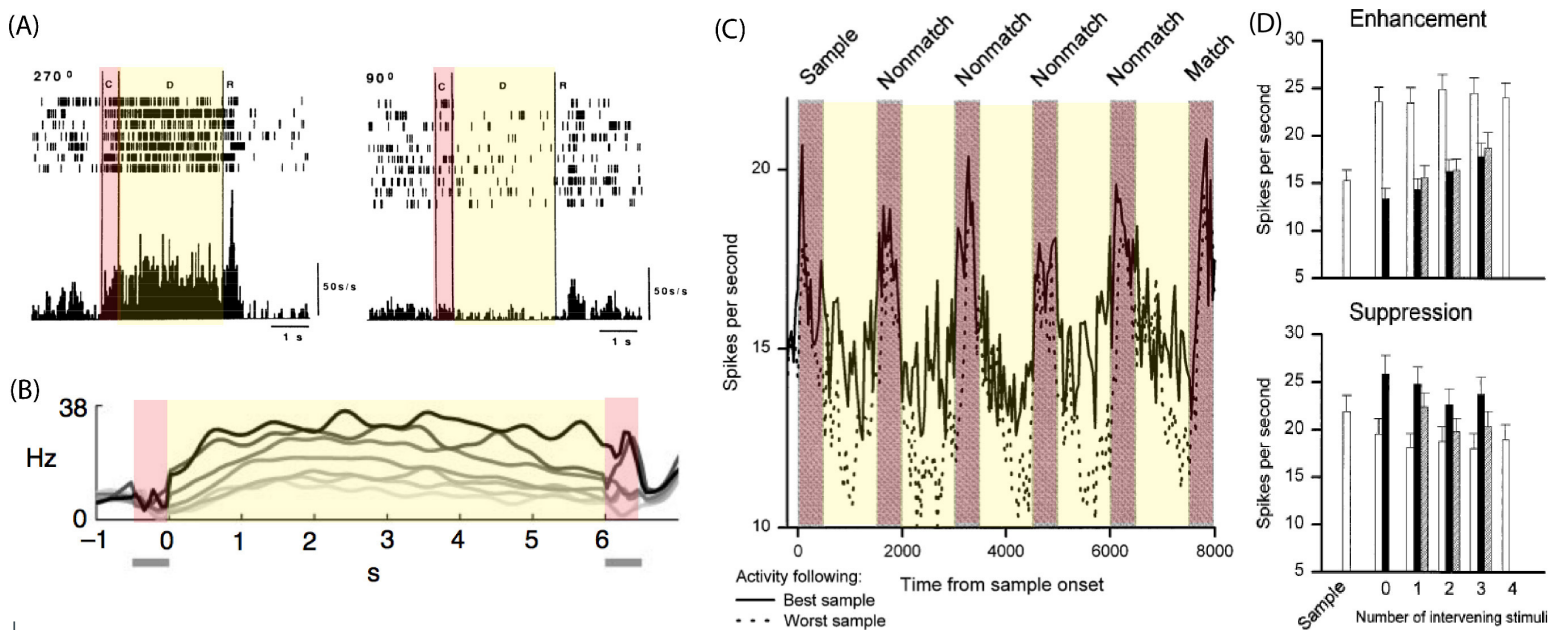
Selective persistent activity in delay-response tasks. Neural activity in different tasks: Yellow and red shaded regions represent delay period and cue presentations respectively. (A) Rastergram (top) and mean firing rate (bottom) of a principal sulcus neuron during an oculomotor delayed-response task for a visual cue presented at 270^o^. (left) and a visual at 90^o^ (right). Adapted with permission from Funahashi et al. (1989); (B) Average activity of one prefrontal neuron during a vibrotactile discrimination task. Different curves correspond to different base frequencies, gray scale from lighter to darker corresponds to increasing frequency values. Adapted with permission from Romo et al. (1999); (C) Averaged activity of prefrontal neurons selective to different sample stimuli. The continuous (respectively dashed) line corresponds to average over trials where the delay activity elicited by a particular sample cue is maximal (respectively minimal) delay activity. Adapted with permission from E. K. Miller et al. (1996). (D) Average response to stimuli that elicited match enhancement (top) and suppression (bottom). Adapted with permission from E. K. Miller et al. (1996).

 Summarizing some of the key results described above, a number of experimental works, now considered as classics in systems neuroscience, showed the necessary and sufficient role of persistent activity in the PFC in WM. During the delay period of an ODR task, the principal sulcus neurons in the PFC show persistent activity which is selective to the sample position cue and correlates with correct task performance (Funahashi et al., 1989). This activity is enhanced only at the preferred angle (see Figure 2a). The activity in the same neurons is suppressed compared to that during the fixation phase when the visual cue is presented at an opposite angle. This persistence does not correspond to the motor preparatory activity preceding the saccade (Funahashi, Chafee, & Goldman-Rakic, 1993). 

 Neurons in the PFC neurons can also have persistent activity in a vibrotactile response task (Romo et al., 1999). The majority of neurons with persistent activity during the delay period have an activity level that depends on the frequency of the base cue (see Figure 2b). Romo et al. (1999) demonstrated that the persistent activity is encoding the base frequency and not the preparation of motor response. A key observation is that while both the ITC (Fuster & Jervey, 1981; E. K. Miller et al., 1996) and PFC neurons’ (E. K. Miller et al., 1996) persistent activity is selective to the sample cue, only in the PFC it is robust to distractors (see Figure 2c), despite the distractor stimuli sometimes causing a sensory PFC response that is stronger than that of the item to be retained (see Figure 2d). 

 These experimental results provide an indication of the underlying dynamics of WM operations. The memory is loaded and maintained by the activation of selective persistent activity, and this persistent activity is robust in the face of distractor presentation. The dynamics of the neurons during the read-out phase suggest that the decision is expressed in the persistent activity of the network (E. K. Miller et al., 1996). Several experiments (Funahashi et al., 1989; Fuster & Jervey, 1981; E. K. Miller & Desimone, 1994; Romo et al., 1999) showed that persistent activity drops abruptly (possibly being actively quenched) around the time of the probe cue presentation when the memory is no longer useful (see Figure 2a). Even though we cannot be certain which command triggers persistent activity’s deactivation, this mechanism suggests that memory is erased by an active instantaneous mechanism rather than by a passive degradation. 

 Several mechanistic hypotheses have been put forth for how persistent activity is generated: based on single cell bistability (e.g., Camperi & Wang, 1998), local network reverberatory activity (e.g., Goldman-Rakic, 1995), or a loop involving several cortical and subcortical areas (e.g., Ashby, Ell, Valentin, & Casale, 2005). In particular, the hypothesis that persistent activity is based on reverberatory excitatory connections is supported by the strong horizontal excitatory connections within a local cortical module (e.g., Gonzalez-Burgos, Barrionuevo, & Lewis, 2000). In section 4, we will review models based both on reverberatory activity (Amit & Brunel, 1997; Brunel & Wang, 2001; Dipoppa & Gutkin, 2013b) and on single cell bistability (Lisman & Idiart, 1995) and discuss their relevance to performance of the operations necessary for WM. Notably, our goal is to propose a functional role for brain oscillatory dynamics in selective gating of WM. 

## Oscillatory Activity in Working Memory

Neural oscillations are commonly defined as a pseudo-periodic rhythmic behaviour generated by individual cells or assemblies. Neural oscillations are ubiquitous in the brain, span a broad frequency range, and emerge both when the brain is performing some cognitive operation and when it is presumably not involved in any specific task. Several alternative theories are available in the literature about the role of neural oscillations, in particular in high-level cognitive processes.

 Brain oscillations have been associated with specific cognitive processes, such as binding (Gray & Singer, 1989), phase-coding (Lisman & Idiart, 1995), spatial coding through phase precession (Jensen & Lisman, 2000; O’Keefe & Recce, 1993), speech parsing (Hyafil, Fontolan, Kabdebon, Gutkin, & Giraud, 2015), support of neural plasticity (Fell & Axmacher, 2011), or gating strategies, such as communication-through-coherence (Fries, 2005). An opposing hypothesis states that oscillations have no functional role and are an epiphenomenon emerging from underlying neural mechanisms, or even an inheritance of the brain circuits that required these features during development or in previous evolutionary stages (Wang, 2010). In this review, we take an alternative position, proposing that oscillations do not in themselves encode or carry information but are instrumental in setting the brain WM circuits in the appropriate dynamic gating modes, allowing for execution of WM operations (Dipoppa & Gutkin, 2013b). 

 Different oscillatory frequency ranges spanning the measurable have been associated with different contexts. This has led to an arguably artificial definition of several frequency bands: delta (1-4 Hz), theta (4-8 Hz), alpha (8-13 Hz), beta (13-30 Hz), and gamma (30-200 Hz) oscillations (e.g., Uhlhaas, Haenschel, Nikolic, & Singer, 2008). Boundaries between the frequency ranges are not equally defined in the literature. Also, some bands have been split into different sub-bands (e.g., Canolty et al., 2006). Virtually every cognitive function has been associated with alterations in the power of several frequency bands, such as, for example, theta, alpha, and gamma bands in WM (Roux & Uhlhaas, 2014). This raises the question of whether is it possible to disentangle whether these oscillations relate to an active and specific process or rather to a generic cognitive state of the brain. 

### Role of Various Oscillation Frequency Bands in Working Memory

 Execution of delayed-response tasks is generally accompanied by either an increase or a decrease in power at different frequencies in humans (Tallon-Baudry et al., 1998) and non-human primates (Pesaran et al., 2002). This oscillatory activity is found in distinct brain areas, such as occipito-temporal cortex for visuo-spatial tasks (Tallon-Baudry et al., 1998), somatosensory cortex for tactile tasks (Haegens, Osipova, Oostenveld, & Jensen, 2010), temporal for audiospatial tasks (Lutzenberger, Ripper, Busse, Birbaumer, & Kaiser, 2002), and frontal cortex for most WM tasks (Haegens et al., 2010; Lutzenberger et al., 2002; Tallon-Baudry et al., 1998). Oscillatory dynamics follow a temporal pattern that correlates with the stages of the task, thereby suggesting a link with WM processing. Induced increases in oscillatory power during WM retention have been detected in the theta (Jensen & Tesche, 2002; H. Lee, Simpson, Logothetis, & Rainer, 2005; Raghavachari et al., 2001; Sauseng et al., 2009; Tesche & Karhu, 2000), beta (H. Lee et al., 2005; Spitzer, Wacker, & Blankenburg, 2010) and gamma (Howard et al., 2003; H. Lee et al., 2005; Pesaran et al., 2002; Pipa et al., 2009; Tallon-Baudry et al., 1998, 1999) rhythms. 

 It remains unclear whether each of these frequency bands can be assigned distinct and specific active roles in WM. For example, after its first discovery (Berger, 1929), the alpha rhythm was assumed to be associated with an absence of active cognitive functions (cortical idling, Adrian & Matthews, 1934). However, in a study showing that alpha power increases with WM load, it was first suggested that alpha power could have an active role in inhibiting information irrelevant for the WM task (Klimesch et al., 1999). The power of alpha oscillations increases in areas encoding irrelevant information relative to areas encoding relevant information (Jensen, Gelfand, Kounios, & Lisman, 2002; Jokisch & Jensen, 2007). This effect is particularly evident in bilateral tasks where the relevant and irrelevant information are segregated in the left and the right hemifield and alpha activity increases in the hemisphere encoding the irrelevant sensory cues (Grimault et al., 2009; Sauseng et al., 2009; Van der Werf, Jensen, Fries, & Medendorp, 2008). Furthermore, Sauseng et al. (2009) have shown that externally induced oscillations in the alpha range can cause effects similar to the physiological alpha rhythm: If such oscillation, induced by repetitive transcranial stimulation (rTMS), is applied to the area encoding the relevant (respectively irrelevant) information, the task performance decreases (respectively increases). 

### Theta Oscillations (4-8 Hz)

**Figure 3.**
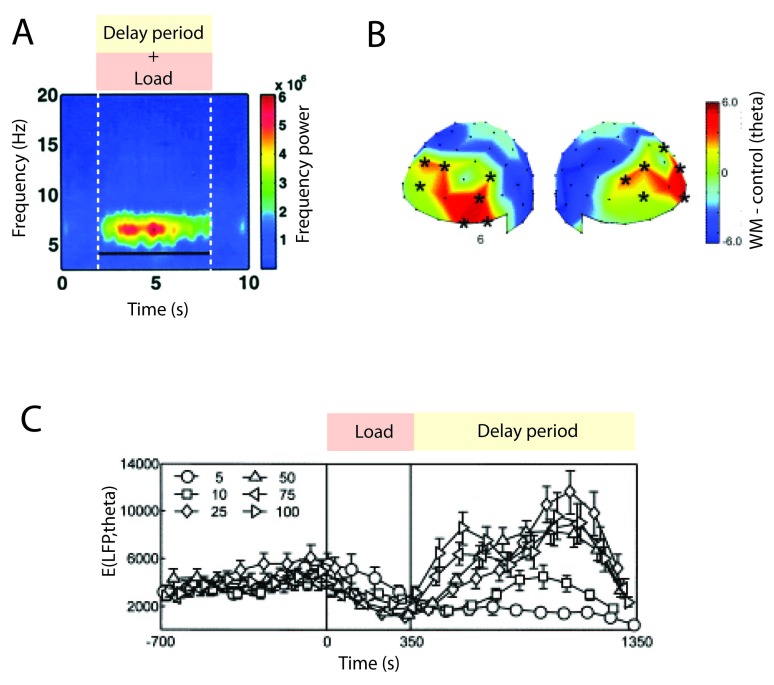
Theta activity associated with memory retention. (A) iEEG recording on epileptic humans performing a verbal Sternberg task. Adapted with permission from Raghavachari et al. (2001). (B) MEG recording on humans performing a Sternberg task. Adapted with permission from Jensen and Tesche (2002). (C) Average theta component of the local field potentials (LFP) signal of a monkey performing a visual delayed match-to-sample (DMS) task for different contrast level of the visual stimulus. The recordings are in the V4 region. Adapted with permission from H. Lee et al. (2005).

 In humans, theta oscillations (4-8 Hz) in humans have originally been associated with recordings of awareness-related brain states (Cobb & Muller, 1954; Daniel, 1967; Dixon & Lear, 1964). The link between theta oscillations and WM was discovered through EEG recordings, first in rats (Landfield, McGaugh, & Tusa, 1972) and then in humans performing an N-back task (Gevins et al., 1997). However, the perception, delay, and decision phases are overlapping, which makes it impossible to distinguish the influence of memory retention from that of encoding and decision. Raghavachari et al. (2001) showed that in iEEG recordings in epileptic patients theta activity increases at the beginning of stimulus presentation in various cortical sites and is sustained during the distinct delay period but drops at probe presentation (see Figure 3a). Hence, theta could be related to memory maintenance and not, at least not exclusively, to loading. Induced theta activity associated with memory retention in the cortex has been observed in non-epileptic patients with MEG recording in humans performing the Sternberg task (Jensen & Tesche, 2002). In particular, Jensen and Tesche (2002) have shown that theta activity is prominent in the PFC and increases parametrically with memory load (see Figure 3b). 

 Memory-induced theta activity has been measured also at the single cell level in electrophysiological experiments in awake monkeys performing the DMS task (H. Lee et al., 2005). To compare with human data showing an increase of theta power in the iEEG in the occipital cortex (Raghavachari et al., 2001), H. Lee et al. (2005) recorded the visual extrastriate (V4) local field potentials (LFP). This LFP signal reflects the aggregate activity of a local area, and it has been closely associated to the iEEG signal. The analysis of LFP and the single unit activity (SUA) signals in V4 neurons shows that theta activity grows in both signals during memory retention (H. Lee et al., 2005; see Figure 3c). This result shows that the increases of theta power in the monkey and the human during the delay period of a WM task are consistent. The experiment by H. Lee et al. (2005) has shown that the LFP signal filtered in the theta band and the spiking activity are phase locked during memory retention. 

### Alpha Oscillations (8-13 Hz)

 Understanding the functional role of alpha oscillations is rather challenging, as they have been associated with various and sometimes contrasting cognitive functions, cognitive states, or neural processes. Alpha oscillations were first found in humans at eye closure (Berger, 1929). It has first been hypothesized that alpha activity is related to cortical idling, a state of an awake subject’s area that is not involved in any specific neural operation (Adrian & Matthews, 1934). This hypothesis was further supported by the contrast with the oscillations: With increasing task difficulty, theta power increases, while alpha power decreases (Gevins, Zeitlin, Doyle, et al., 1979; Gevins, Zeitlin, Yingling, et al., 1979; Gundel & Wilson, 1992). 

 Subsequent experiments have associated this band with more active processes, such as functional inhibition (Klimesch et al., 1999; Klimesch, Sauseng, & Hanslmayr, 2007), temporal framing (Palva & Palva, 2007; VanRullen & Koch, 2003; Varela, Toro, John, & Schwartz, 1981), attentional modulation (Bollimunta, Mo, Schroeder, & Ding, 2011; Mo, Schroeder, & Ding, 2011), cross-modal binding (e.g., Hummel & Gerloff, 2005), or mental calculation and imagery (e.g., Cooper, Burgess, Croft, & Gruzelier, 2006). It has been proposed that the different functions connected to a change in alpha power can be explained by event-related alpha desynchronization linked to active processing, such as retrieval in WM tasks, as opposed to alpha synchronization linked to functional inhibition of irrelevant information required during retention in WM tasks (Klimesch et al., 1999). In fact, alpha oscillations are a common dynamic in the cortex: Primate experiments have shown that alpha oscillations are found across all cortical depths in multiple brain areas (Bollimunta, Chen, Schroeder, & Ding, 2008; Haegens et al., 2015), giving further potential support to the link with cortical idling. 

**Figure 4.**
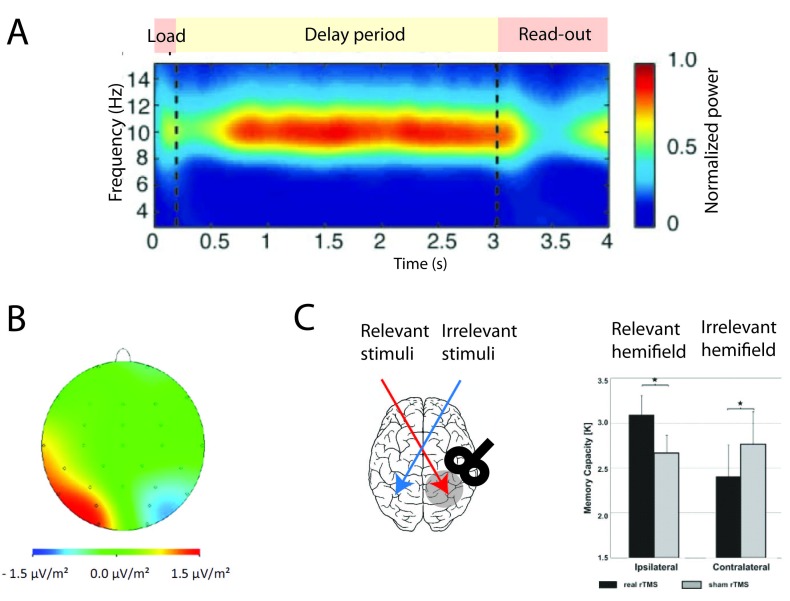
Alpha activity in WM task. (A) Time-frequency analysis EEG signal in human performing a verbal Sternberg task. The time-frequency power is averaged for different trials and number of encoded items, in sites with a significant increase in alpha during retention. Adapted with permission from Jensen et al. (2002). (B) Average EEG signal in a human performing a bilateral visual delayed response task during retention. Relevant stimuli are presented to the left and irrelevant stimuli are presented to the right hemifields. Topographic maps of alpha amplitude differences between “left items retained” and “right items retained”. Adapted with permission from Sauseng et al. (2009). (C) Same task as described in (B). In the left: outline of the experiment. rTMS at 10 Hz is applied to the posterior parietal cortex (gray shaded area). In the plot on the right, black bar represents rTMS applied to respectively the posterior parietal cortex ipsilateral and contralateral to the relevant stimulus, respectively. Gray bars represent a control condition. Adapted with permission from Sauseng et al. (2009).

 Several experiments have shown that alpha activity may also be associated with active functions. For example, in humans performing delayed response tasks, alpha activity increases during memory retention and drops at task completion, as measured in the EEG signal (Busch & Herrmann, 2003; Jensen et al., 2002; Sauseng et al., 2005). In the posterior and in the bilateral central areas of subjects performing the Sternberg task, the alpha activity increases in power (Jensen et al., 2002; see Figure 4a) and, more importantly, increases with memory load. 

 The results of the study by Jensen et al. (2002) at first glance contrast with the idling hypothesis, since alpha activity increases with memory load and thus with cognitive effort. The active processing hypothesis is further reinforced by other experiments showing that alpha activity increases with cognitive demand, such as during conscious somatosensory perception (Palva, Linkenkaer-Hansen, Näätänen, & Palva, 2005) and mental imagery (Cooper et al., 2006). There are two possible explanations for this increase: Either alpha oscillations are involved in active processing within the brain circuits carrying relevant WM information (Palva & Palva, 2007), or they are involved in active inhibition of areas carrying irrelevant information (Jensen et al., 2002). 

 Further evidence for the inhibition hypothesis originates from bilateral visual delayed response tasks where relevant stimuli are presented in one of two visual hemifields (left or right) of a screen (Grimault et al., 2009; Sauseng et al., 2009; Van der Werf et al., 2008). The subject should maintain in memory these relevant stimuli that are thought to be processed in the contralateral hemisphere (Funahashi, Bruce, et al., 1993). In these experiments, alpha activity increases in the hemisphere ipsilateral to the relevant stimuli (see Figure 4b). In particular, in the experiment presented by Sauseng et al. (2009), irrelevant stimuli are also presented in the hemifield opposed to that containing relevant stimuli. Alpha activity, measured by the EEG signal, increases as a function of the number of irrelevant items presented, consistent with the inhibition hypothesis. 

 Additionally, if rTMS at 10 Hz is applied to the parietal area ipsilateral to the irrelevant stimuli (that encodes relevant information), then working memory capacity decreases (Sauseng et al., 2009 ; see Figure 4c). This can be explained by alpha disrupting correct memory formation (Sauseng et al., 2009). If instead rTMS is applied to the parietal area contralateral to the irrelevant stimuli, then working memory capacity increases. This could be explained by alpha impeding the irrelevant stimuli from interfering with the neural representations of the memorized relevant items. Additionally, if the applied rTMS has a frequency of 15 Hz, hence is outside of the alpha band, there is no significant effect on task performance, showing that this oscillation-induced inhibition is intrinsically related to alpha oscillations. 

 As we mentioned above, functional inhibition is not a process unique to WM but is also required in other contexts as, for instance, visual attention. Lateralization of alpha activity also emerges during visual attention and sensory discrimination tasks (Haegens, Luther, & Jensen, 2012; Händel, Haarmeier, & Jensen, 2011; Van der Lubbe & Utzerath, 2013; Worden, Foxe, Wang, & Simpson, 2000). The same holds true for rTMS experiments during a visual detection task: If an alpha oscillation is applied in the occipital or parietal cortex of the hemisphere contralateral to a stimulus, the performance decreases. Since an external alpha oscillation influences neural processing, we may be led to argue that the functional inhibition could be based on a top-down modulation originating from an external executive brain area and that we are reproducing this effect with an rTMS artificially. Top-down modulation by alpha oscillations is plausible in light of long-range synchronization detected by MEG, for example, between prefrontal and parietal cortex (Grimault et al., 2009). 

 While the inhibition hypothesis may be reconciled with the idling, as in both information is arguably prevented from entering active processing, a key unsolved issue is what could be the neural mechanism underlying this functional inhibition. It has been proposed that alpha-induced functional inhibition corresponds to rhythmic synaptic inhibition of neurons across all cortical layers. This rhythmic inhibition would be generated by local interneuronal circuits in the deep layers during a decrease of thalamic excitatory drive ( Womelsdorf, Valiante, Sahin, Miller, & Tiesinga, 2014). It was found that gamma synchronization is modulated by the phase of a concurrent alpha oscillation (Osipova, Hermes, & Jensen, 2008; Voytek et al., 2010). This might support the hypothesis that alpha-induced functional inhibition corresponds to synaptic inhibition, since gamma synchronization is linked to activity of local neurons and notably GABAergic interneurons, while alpha oscillation can be generated outside the local cortical circuit. An alternative proposal is that alpha oscillations are able to deactivate a persistent state. In dynamical systems terminology, this deactivation corresponds to driving the persistent state out of its basin of attraction by directly inducing excessive spiking synchronization within the persistent activity, rather than by inhibiting the system (Dipoppa & Gutkin, 2013b). Note that this synchronization can be induced by excitatory effects of alpha-locked synaptic inputs to the local circuit neurons or from inhibitory synapses (Van Vreeswijk, Abbott, & Ermentrout, 1994). 

### Beta Oscillations (13-30 Hz)

 Beta rhythm is thought to be involved in WM maintenance (Tallon-Baudry et al., 1999) and, in addition, in motor preparation (Murthy & Fetz, 1992), holding fixed motor positions (Kilner, Baker, Salenius, Hari, & Lemon, 2000), and sensory gating of salient and novel stimuli (e.g., Hong, Buchanan, Thaker, Shepard, & Summerfelt, 2008). When the memory trace needs to be actively maintained, induced beta activity during the WM delay period is measured in monkeys (Tallon-Baudry, Mandon, Freiwald, & Kreiter, 2004) and humans (Spitzer et al., 2010; Tallon-Baudry et al., 1999). 

### Gamma Oscillations (30-200 Hz)

 In addition to WM retention, gamma rhythm has been associated with attention (e.g., Makeig & Jung, 1996) and integration of sensory information (Gray, 1994). Induced gamma activity also increases during WM retention, as measured in monkeys (Pesaran et al., 2002) and humans (Howard et al., 2003; Kaiser, Ripper, Birbaumer, & Lutzenberger, 2003; Lutzenberger et al., 2002; Tallon-Baudry et al., 1998, 1999). In particular, by recording the iEEG signal in epileptic human subjects, Howard et al. (2003) found that the power of gamma increases with memory load. Furthermore, the number of items (Roux, Wibral, Mohr, Singer, & Uhlhaas, 2012) and the information content (Polania, Paulus, & Nitsche, 2012) held in WM can be predicted from the single trial fluctuations of gamma oscillations in non-invasive recordings of the human PFC. 

 The description of these bands is further complicated by their subdivision in sub-bands that have different properties during cognitive tasks and, in particular, in delayed response tasks. For example, Pipa et al. (2009) have found that only the high gamma sub-band is predictive of the correct task performance during the whole delay period in a WM task. 

### Computational Models of Working Memory and Implementation of Gating Dynamics

 Numerous theoretical models have been proposed to describe the manipulation of the STM in the WM system. Most of these are based on a paradigm in which different memories are encoded by different attractor states (Hebb, 1949) corresponding to stable neural activity patterns. If the system can be described by a set of dynamical equations describing its evolution, then an attractor corresponds to a set of solutions of those equations toward which the dynamical variables converge in time. For example, if one of the variables of the equations is the population firing rate, then an asynchronous network attractor state would correspond to a stationary point and an oscillatory state would correspond to a stable limit cycle. The neural structure sustaining attractor states could be a pre-existing columnar organization (e.g., Goldman-Rakic, 1995) or could be generated by long-term plasticity (e.g., Hebb, 1949). 

 The nature of the attractor state further defines the type of models and mechanistic hypotheses for how WM is manipulated and gated during task execution. Models in which STM content is encoded by asynchronous persistent activity (the attractor state) have been able to describe discrete-item WM (e.g., Amit & Brunel, 1997; Brunel & Wang, 2001), spatial WM ( Compte et al., 2000; Gutkin et al., 2001; Laing & Chow, 2001), and parametric WM (Machens et al., 2005). In these models, the persistent activity is typically sustained by recurrent connections. Models in which STM content is encoded by nested oscillations of different frequencies (e.g., Lisman & Idiart, 1995) have been able to explain psychophysical results (e.g., the reaction time) from humans performing the Sternberg task (Sternberg, 1966). In these models, the nested oscillations are typically sustained by intrinsic cell properties (e.g., Lisman & Idiart, 1995). 

 Instead of giving an exhaustive list of the various (and at times mutually incompatible) WM models, we will highlight how these theoretical proposals have dealt with WM gating and operations. Several alternative theoretical proposals have been made where WM content is encoded by a persistent calcium buffer and maintained through synaptic plasticity (Mongillo, Barak, & Tsodyks, 2008), selective temporal organization of oscillatory activity (Kopell et al., 2011; Szatmary & Izhikevich, 2010), or intrinsic bistability of the PFC modules in a large scale model requiring reverberation between multiple cortical and subcortical structures (Frank et al., 2001; we note that PFC bistability may in fact be a result of persistent activity mechanisms at the level of local neural circuits). In this review, we specifically focused on local attractor models of WM maintenance, since these are supported by a large number of experimental data (collected across animals and experiments over the last 30 years), are prevalent in the computational neuroscience literature, and their success to make predictions that are compatible with the data (Riley & Constantinidis, 2015; Wimmer et al., 2014). 

 Most of the models belonging to these three classes (WMAS sustained by recurrent connections, single cells bistability, or synaptic plasticity) are based on a similar gating mechanism (i.e., the control of which operations need to be executed based on the context), namely, the recruitment of inhibitory interneurons. In particular, several influential computational models are based specifically on modulations of inhibition in the local cortical WM active storage network (e.g., Brunel & Wang, 2001; Compte et al., 2000). We will describe first the inhibition-induced gating and then contrast it with the recently proposed alternative mechanisms, such as oscillation-induced gating.

### Inhibition-Induced Gating as a Mechanism for Working Memory Operations

 The inhibition-induced gating paradigm corresponds to controlling the encoding and maintenance of memory using a temporally focused global inhibition. By *global* we mean inhibition that projects non-selectively to all the sub-circuits in the WM active storage network. One experimental finding that has been leveled as support for such global inhibitory process is that during the delay period, the activity of neurons selective to stimuli different from the sample cue is suppressed compared to their spontaneous activity during fixation (Funahashi et al., 1989). We can understand this paradigm through two different examples of WM belonging to the previously mentioned classes, namely: memory encoded by asynchronous reverberatory persistent activity (Amit & Brunel, 1997; Brunel & Wang, 2001) and oscillatory persistent activity (Lisman & Idiart, 1995). 

### Gating in Discrete-item Working Memory Models Based on Persistent Activity

 Amit and Brunel (1997) introduced a local spiking network that can memorize discrete items and reproduce electrophysiological data (e.g., Fuster & Jervey, 1981). The network includes a finite number of selective excitatory populations and one inhibitory population. Depending on the amount of average input received, each population can be in a state where all its neurons are either in a subthreshold regime, corresponding to a baseline state, or in a suprathreshold regime, corresponding to a persistent state. 

Within a certain range of synaptic strengths, the network acts as a “winner-take-all” system with a finite number of attractor states: Either one and only one of the selective excitatory populations is in a persistent state while all the other populations are in the baseline state (equal to the temporary storage of the discrete item), or all the populations of the network are in the baseline state (absence of any stored memory). A selective population can be activated by an excitatory sample stimulus. Hence this network includes the operations load and maintain.

**Figure 5.**
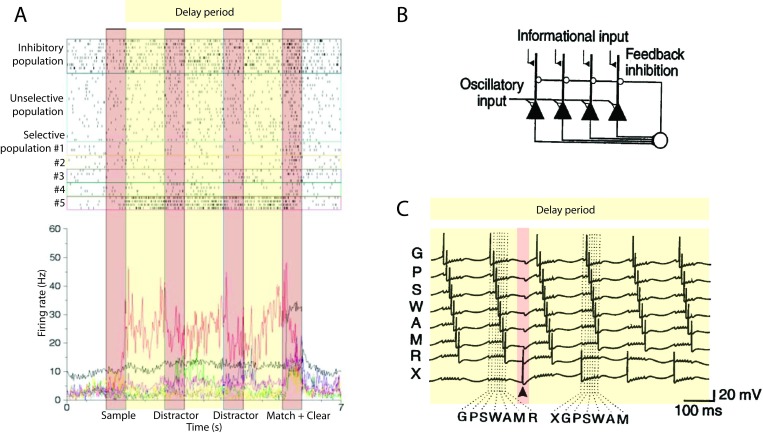
A) Discrete-item working memory based on feedback inhibition. Rastergram (top) and average firing rate (bottom) of the network performing a delayed matching-to-sample task for weak distractors. Red shaded areas represent stimuli presentation and yellow shaded area represent delay period. Sample (red trace), first distractor (green trace), second distractor (violet trace) and match (red trace) stimuli have the same strength, but only the population excited by the sample (red) access the persistent state. The response of populations excited by distractors (green and violet) is weak because of the increased nonspecific inhibition. At the end of the task, a non-specific clear stimulus (blue) erases the activity in all the network. Adapted with permission from Brunel and Wang (2001). (B) Multi-item working memory models based on oscillations. Outline of the network: Pyramidal neurons project axons to a global feedback inhibition. Adapted with permission from Lisman and Idiart (1995). (C) Each neuron encodes a different memory. The neurons emit spikes in sequence, each in a separate gamma subcycle, nested in a slower theta cycle. The network cannot sustain more than seven memories. If an additional memory, “X”, is encoded another one, ”R”, is erased. Adapted with permission from Lisman and Idiart (1995).

 Brunel and Wang (2001) introduced a spiking network that implemented the operations of prevent and *clear* (see Figure 5a). These authors thus partly reproduced the pattern of activity obtained by E. K. Miller et al. (1996) in the PFC: Non-matching stimuli increased transiently the activity in their corresponding non-relevant selective populations but failed to activate the persistent state in those populations (see Figure 5a). The WM model is, therefore, capable of preventing the activation of persistent activity in the non-relevant populations by the distracting stimuli. This feature is due to the winner-take-all effect created by the global nonspecific inhibition. However, unlike in the results of E. K. Miller et al. (1996), the transient activity induced by the distractors must not increase beyond the level of activity in a persistent state and must be below the corresponding response to the loaded item, otherwise the model would incorrectly memorize the distractor item and remove from memory the sample item. 

 In the model of Brunel and Wang (2001) the operation clear is executed by a global excitation directed to all the populations of the network, and it putatively originates from a reward or motor area. The strength of the *clear* excitatory signal is such that activity in the inhibitory population is increased more than in the excitatory populations. In this model, the recurrent synaptic connectivity between the various neural populations needs to be exquisitely balanced to capture the active memory storage, the cross-item suppression (without loss of the memory), and the operation of clear by the net inhibitory transients. 

### Inhibition-induced Gating in Models of Spatial Working Memory

 In attractor-based models of spatial WM, the attractor state must encode the spatial position. If the spatial measure is a periodic one-dimensional continuous variable (such as orientation), the encoding can be achieved by neurons with different receptive fields forming a ring in the abstract space of connections (Compte et al., 2000). The memory of a spatial position is encoded by an attractor state called *bump state*, where the neurons centered at the preferred position (orientation or an angular position of the stimulus in the visual field) corresponding to the memory location fire persistently at the maximal rate in the network and surrounding neurons fire in a decreasing amount the farther away they are from the centered neuron. 

 A network that can sustain such a bump state has excitatory-to-excitatory synaptic strengths that depend on their mutual distance, while the inhibitory neurons have no or wider dependence on distance (Compte et al., 2000). This results in a lateral inhibition, or Mexican-*hat* networks, that can sustain a bump state together with a nonspecific low-firing steady state that corresponds to the spontaneous state. Lateral inhibition prevents the spread of activity to cells outside the focus of the bump state. 

 In the model of Compte et al. (2000), the operations of prevent and clear are executed through the recruitment of inhibitory neurons, as in the WM model of Brunel and Wang (2001). The bump state activated by the sample increases the overall inhibition that in turn prevents the activation of a new bump state by a distractor. Also, in this case, the prevent operation fails upon presentation of distractors that are too strong. The excitatory signal of clear causes a global increase of both excitatory and inhibitory activity. However, the network is tuned such that the inhibition dominates and deactivates the bump state. 

### Gating in Oscillatory Nesting Model for Multi-item Working Memory Maintained via Temporal Segmentation

As described in section 3, a vast experimental literature shows, both at a macroscopic and microscopic scale, that WM is accompanied by neural oscillations. Several models have provided useful insights in this debate by showing how these oscillations can have a crucial functional role in WM at the level of coding or neural dynamics.

 The earliest WM model based on oscillations was introduced by Lisman and Idiart (1995) to describe the encoding of up to seven items in accordance with psychophysical data (G. A. Miller, 1956). Information is multiplexed by nesting low-frequency oscillations (around 6 Hz) with high-frequency oscillations (around 40 Hz). The two frequencies lie in the theta band (4-8 Hz) and the gamma band (30-100 Hz) respectively. 

The model of Lisman and Idiart (1995) consists of a network of excitatory pyramidal neurons connected with inhibitory interneurons. Each pyramidal neuron receives subthreshold theta oscillatory input. The emission of a spike by a pyramidal neuron induces a ramping afterdepolarization (ADP). The elevated membrane potential of the ADP leads the pyramidal neuron to fire during the subsequent theta cycle. Therefore, each neuron is intrinsically bistable. The activation of each pyramidal neuron by an excitatory input represents the encoding of a distinct item in memory.

 The pyramidal neurons form a disynaptic loop together with global inhibitory interneurons that cause feedback inhibitory post-synaptic potentials that are alpha-pulses (see Figure 5b). Therefore, the emission of a spike by a pyramidal neuron increases the overall level of inhibition. The model was extended by Jensen, Idiart, and Lisman (1996) and Jensen and Lisman (1996), so that a memory is encoded by selective populations of pyramidal neurons, with the synaptic strengths potentiated within the same selective population. The increasing reaction time is a function of the number of items encoded (Sternberg, 1966) suggesting that the information read-out is processed through a serial scanning of the item sequence. 

Lisman and Idiart (1995) proposed that the information is erased by replacing old memories with new memories, as opposed to the previous mechanisms, where there is an explicit top-down modulated operation of clear (see Figure 5c). In fact, once the network of Lisman and Idiart (1995) has encoded seven items in memory, any additional item to be encoded will deactivate the persistent activity of the last item in the sequence. Indeed, the additional inhibition shifts the phase of all the memory items in the theta cycle. Since the persistent activity is deactivated by a decrease of excitation (equivalent to an increase of inhibition), we can include this mechanism in the inhibition-induced gating framework. As far as we know, in this model and its variants, the operation of prevent is not explicitly implemented and, in the absence of any additional specific mechanism, a distractor could activate the corresponding selective population.

In summary, both the two classes of spatial and discrete WM models we presented above rely on inhibitory synaptic influences for selective gating. In the attractor model paradigm, the carefully adjusted global inhibition implements a conditional winner-take-all mechanism to select item versus distractor to globally suppress the memory trace. In the oscillatory nesting case, any item encoded after the memory capacity has reached its limit will increase the inhibition level through a disynaptic loop and remove a previously encoded memory.

Interestingly, the inhibition-induced gating implies necessarily that the transient responses evoked directly by the stimulus should be lower for the distractor than for the item to be stored in working memory. This relative suppression of the sensory response is a necessary network signature of selective suppression of the distractor. In cases where the distractor produces a stronger transient response than the item, the suppression fails and the models fail to complete the task correctly.

### Synchronization-induced Gating

 In inhibition-induced gating models, the operation of clear arises from an increase of inhibition. An alternative model proposes that this operation arises from an increase in the degree of synchronization (Gutkin et al., 2001; Laing & Chow, 2001). The corresponding spatial WM model is aimed at reproducing electrophysiological results from the ODR tasks (Funahashi et al., 1989), and has a spatial structure similar to another WM model where gating was based on inhibition ( Compte et al., 2000). 

 Experiments supporting this model have shown a behaviour-dependent modulation of the level of spike synchronization during WM tasks (Pipa & Munk, 2011; Sakurai & Takahashi, 2006). When we refer to synchronization, we should distinguish between *phase synchronization* (two periodic signals oscillate with a constant phase difference) and *spike synchronization* (spikes from different neurons are emitted within a short time window). Assemblies of neurons can emit synchronous spikes depending on the task or the stage of the task (Sakurai & Takahashi, 2006). This suggests that there is an underlying functional connectivity modulating the spiking activity in WM. During most of the delay period, the synchronized events are more frequent and have more cells involved in trials performed correctly than in trials performed incorrectly (Pipa & Munk, 2011). In another study, in trials where a monkey performed a WM task correctly, there was a significant increase of coupling between lower gamma oscillations and local spikes during the test cue presentation, hence a sign of increased spike synchronization at the period in which the memory needs to be cleared (Wu, Wheeler, Staedtler, Munk, & Pipa, 2008). 

### Spatial Working Memory Based on Synchronization Gating

 Along with the model of Compte et al. (2000), Gutkin et al. (2001) and Laing and Chow (2001) implemented a spatial WM network where fast Alpha-amino-3-hydroxy-5-methyl-4-isoxazolepropionic acid (AMPA) synapses are sufficient to maintain a bump state (and thus the slow N-methyl-D-aspartate [NMDA] synapses are not necessary). Differently from the model of Compte et al. (2000), the bump state is deactivated through the synchronization of the spike times, and not by inhibition. This mechanism is based on the fact that the persistent bump needs asynchronous activity (and hence recurrent synaptic inputs to each neuron) to be maintained. In fact, neurons have an intrinsic refractory period during which they are unable to emit a spike. If all the neurons are synchronized, then the neurons receive all their recurrent input immediately after they have emitted a spike. Since the bump state is sustained by recurrent connections, at that moment the input is not effective because of the refractoriness, and the bump state is disrupted. 

 In conclusion, the models of Gutkin et al. (2001) and Laing and Chow (2001) show that another paradigm, based on synchronization rather than inhibition, can underlie the gating mechanism. This alternative gating can lead to a fast operation clear since the mechanism is monosynaptic. However, some aspects are not addressed in this model. The first question is whether or not it is possible, with a synchronization-induced gating, to have a context-dependent use of sensory stimuli in order to perform all the operations in the absence of further additional control stimuli, as in the model of Machens et al. (2005). The second question is whether the prevent operation could also be executed through a mechanism involving synchronization of the spike times, despite the recruitment of inhibition. 

### Synchronization-based Gating in Discrete Working Memory.

 In a discrete WM model, Dipoppa and Gutkin (2013a) have shown that the prevent operation can be executed by a synchronization-induced gating mechanism. The stability of persistent activity can be modulated on-line not only by the information-related signal, as in Gutkin et al. (2001) and Laing and Chow (2001), but also by the correlation structure of background activity. The basis of this paradigm is that correlations in the background neural activity influence the transition between the persistent state and the quiescent state (Dipoppa & Gutkin, 2013a). 

 The computational WM model based on background correlations is a winner-take-all network composed of two excitatory populations and one inhibitory population. Each of the excitatory populations receives background input from independent stochastic sources and an additional stochastic source common to the neurons of such populations. This is a putative model of ongoing brain activity impinging on the WM system, and not directly modulated by the task demands. The amount of correlation could be modulated independently in the two excitatory populations. In this model, synaptic connections mediated by the fast AMPA receptors are sufficient to maintain the memory, as opposed to Brunel and Wang (2001), where slow NMDA receptors are strictly necessary for this purpose. 

 The key feature of the model is that, by increasing the level of background activity correlations in the populations encoding irrelevant information, the dynamics of the system prevent a distractor from being loaded into the WMAS. In particular, this model can prevent strong distractors more efficiently than in a null model based on inhibition gating. The model can, therefore, reproduce the property of the response to the distractor stimulus being as strong as (or even stronger than) that to the sample stimulus (as in E. K. Miller et al., 1996), an effect not compatible with inhibition-induced gating. An additional feature of this paradigm is that, similarly to Gutkin et al. (2001) and to Laing and Chow (2001), the presentation of the match stimulus can directly erase the memory thereby implementing a direct match-based suppression without requiring inhibition. 

### Oscillation-induced Gating

 Experimental results associate WM with both persistent neural activity and neural oscillations in specific bands (as reviewed above). Pioneering models have based loading and maintaining WM memory on sustained neural oscillations ( Kopell et al., 2011; Lisman & Idiart, 1995). However, these models do not address the key issue of selective gating—that is, how the system can (1) prevent activation by distractors while maintaining the memory trace; and (2) erase an obsolete memory. This issue has been addressed in an alternative model where selective gating is obtained by the control of background oscillations (Dipoppa & Gutkin, 2013b). 

### Flexible Control of Oscillations as a Unified Cortical Circuit Mechanism to Execute Working Memory Tasks.

 The model proposed by Dipoppa and Gutkin (2013b) combines a spiking network WMAS, inspired by the model of Amit and Brunel (1997) and of Brunel and Wang (2001), with background activity modulated by controlled oscillations. In this model, the externally driven oscillations set the WMAS into distinct gating modes, each defined by the oscillation frequency. By shifting the gating modes as a function of the task phase, the WM system successfully performs all the operations of a DMS task with distractors. 

**Figure 6.**
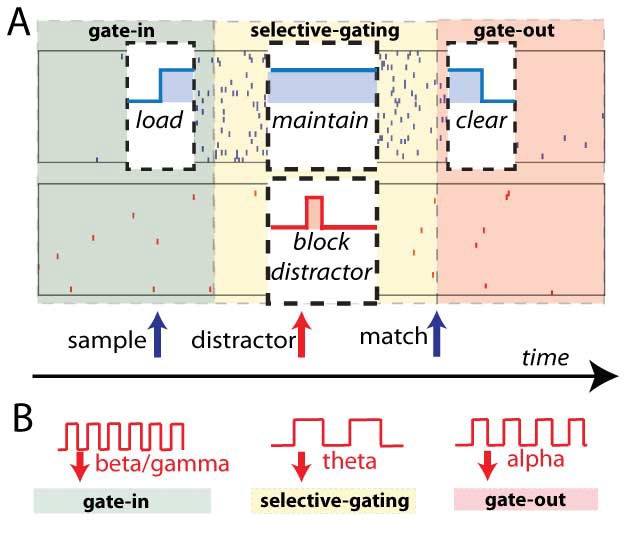
(A) Outline of the delay match-to-sample task with distractors and the required operations with the underlying gating modes. The two rastergrams represent two populations B (in blue) and R (in red). The phases of operations are outlined in a white box showing the succession of the gating modes and operations. Gate-in mode: The sample stimulus (blue arrow) activates population B (load). Selective-gating: The distractor stimulus (red arrow) is not able to activate population R persistently (block distractor) and the memory in population B is held (maintain). Gate-out: Upon match-stimulus presentation, persistent activity is deactivated in the blue population (clear). (B) Outline of the gating modes. Input oscillations enabling the gating modes: Beta–gamma band ensures the gate-in mode at the beginning of the task, theta band ensures the selective-gating mode during the delay period (memory maintenance together with protections from the distractors), and alpha band ensures the gate-out mode at the task completion (memory is rapidly cleared).

 Furthermore, in the WM-with-oscillations model, each different band is associated with a gating mode (Dipoppa & Gutkin, 2013b) that is consistent with experimental studies on oscillations in WM reviewed in section 2. The mechanism for gating is based on external oscillations determining the transitions between the resting state and persistent state. The frequency of the oscillation modulates the probabilities of these transitions, thereby determining three gating modes (see Figure 6a): gate-in, ensuring that a memory item can be loaded and maintained; selective-gating, ensuring that a given preloaded memory item can be maintained but no de novo items can be loaded; and gate-out, where memory can neither be loaded nor maintained. The model shows that the frequency ranges supporting the gating modes are: beta and gamma for the gate-in mode, theta for the selective-gating mode, and alpha for the gate-out mode (see Figure 6b). 

**Figure 7.**
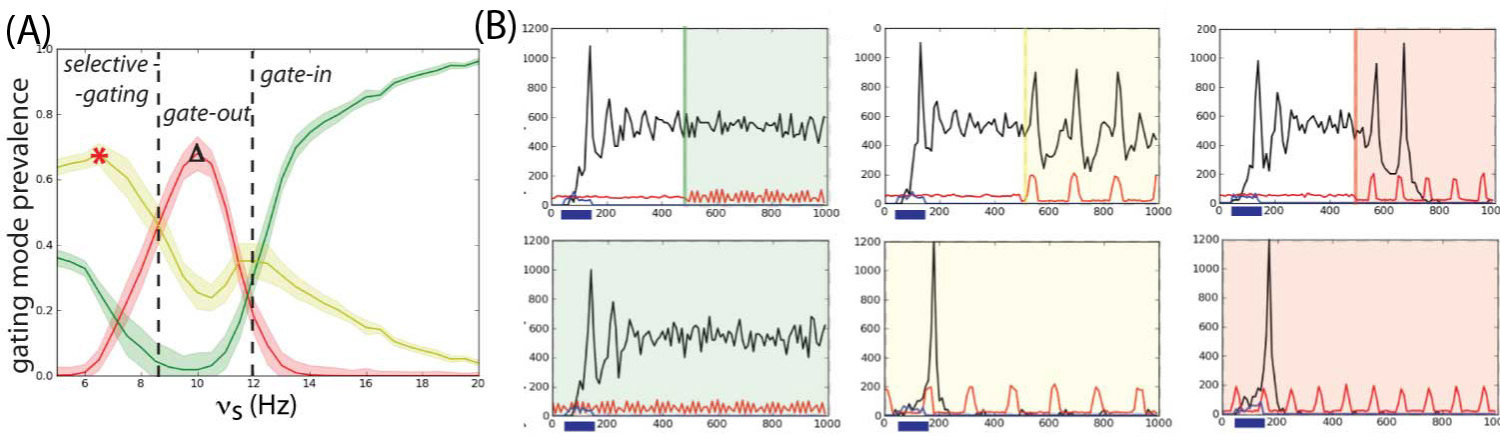
Outline of gating modes. (A) The probability of the gating modes determined by the joint probability: of erase and block (red line), not-erase and not-block (green curve), and not-erase and block (yellow curve). The gate-out mode has maximal value at ν_s_ = 10 Hz (red star). The selective-gating mode has maximal value at ν_s_ = 6.5 Hz (black triangle). The filled space around the curves represents SEM. (B) The network responses to a transient excitatory external stimulus (*t* = 50–150 ms) depend on the frequency content of the background oscillatory input. Plots show average population input from recurrent connections (black), background activity (red), and external stimulus (blue) in arbitrary/normalized units. For each frequency, the background oscillation is switched on either after (top) or before (bottom) the stimulus presentation. Left: Beta–gamma-band oscillations (45 Hz) are compatible with persistent state maintenance. Neither erasing nor blocking is seen. Center: Theta-band oscillations (6.5 Hz) maintain an a priori persistent state while blocking de novo activations. Right: Alpha-band oscillations (10 Hz) inhibit persistent activity: The persistent state is deactivated by oscillations onset and is prevented from being activated by the transient stimulus. Adapted with permission from Dipoppa and Gutkin (2013b).

The relationship between frequency bands and gating modes is illustrated in a single unit network, where the probabilities of occurrence of the gating modes are measured across frequencies (see Figure 7a). The curves have been measured by computing the transition from the persistent state to the resting state and vice versa (see Figure 7b).

**Figure 8.**
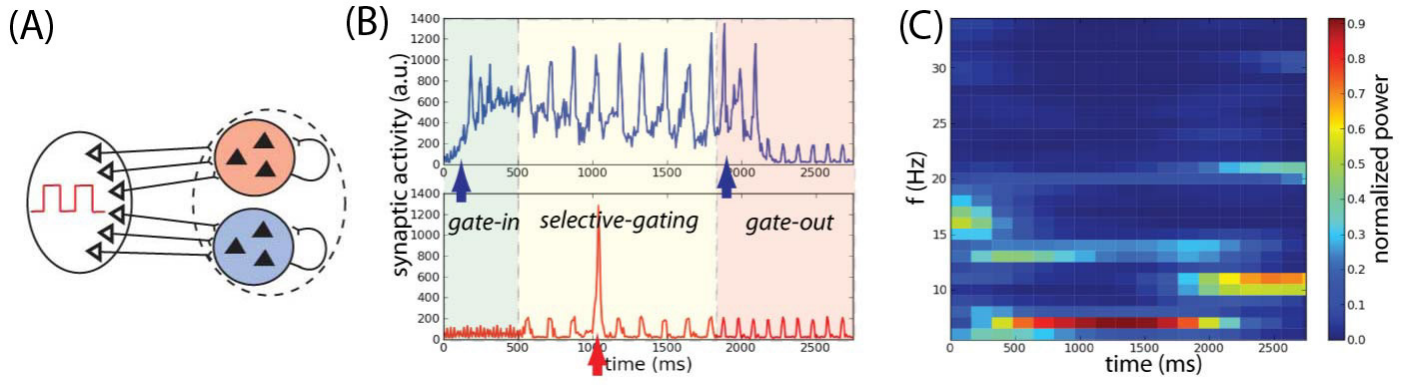
Flexible frequency control of shared oscillations implements the sequential requirements of working memory within a local network. (A) Local two-population unit network: Populations B and R receive input by sources modulated by the shared background oscillation. (B) Average synaptic input simulating a local-field potential of a network performing correctly the delayed matching-to-sample task of the population B (upper panel) and population R (lower panel). Colored areas represent different values of oscillations frequency: gamma band ν_s_ = 45 Hz (green), theta band ν_s_ = 6.5 Hz (yellow), and alpha band ν_s_ = 10 Hz (red). (C) Time-frequency power spectrum of the average synaptic input summed for populations B and R. Adapted with permission from Dipoppa and Gutkin (2013b).

 Building on the oscillation-induced gating paradigm, Dipoppa and Gutkin (2013b) proposed two potential implementations of WM networks. The first model corresponds to a local multi-item spiking network, designed to model WM tasks where all the items are represented in the same area of the prefrontal cortex. In this version of the model, the populations receive a common background input oscillation (see Figure 8a). Flexibly varying the frequency of the oscillation as the demands of the WM unfold in time enables the WM system to perform successfully all the required operations (see Figure 8b). In particular, increasing the theta oscillations during the delay period allows selective memory maintenance, meaning maintenance of the item-memory and protections from distractors (see Figure 8c), which is consistent with experimental observations (Jensen & Tesche, 2002; H. Lee et al., 2005). Furthermore, this model predicts a decrease of alpha power in the WMAS during both the encoding and maintenance phase that is consistent with a study showing such a decrease in the frontotemporal cortices (Heinrichs-Graham & Wilson, 2015). Intriguingly, this study shows that in sensory areas, such as the occipital cortex, alpha power decreases during the encoding phase but increases during the maintenance phase, potentially supporting the idea that an area that does not encode temporary information in the form of persistent activity during the delay period is set in the gate-out mode. 

**Figure 9.**
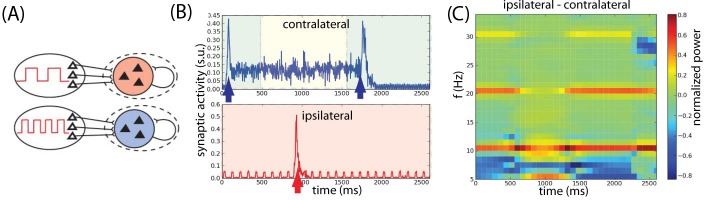
Working memory task execution in a bihemispheric network. (A) Bihemispheric two-population unit network: Populations B and R receive input by sources modulated by independent background oscillations. (B) Simulated local field potentials (LFP)(in synaptic units) of a network performing the delayed match-to-sample (DMS) task correctly: The upper panel gives the LFP of the population B (contralateral) that shows persistent activity turned on by the stimulus and turned off by a second presentation of the same stimulus (match) and the lower panel depicts the LFP of the population R (ipsilateral) showing that the lateralized distractor causes only a transient network response and no persistent activity. Colored area represents different values of oscillation frequency; gamma: ν_s_ = 45 Hz (green), theta: ν_s_ = 6.5 Hz (yellow), and alpha: ν_s_ =10 Hz (red). (C) The difference in the time-frequency power spectrum of the average synaptic activity between the ipsilateral (population R) and contralateral (population B) populations. Adapted with permission from Dipoppa and Gutkin (2013b).

 In the second model, Dipoppa and Gutkin (2013b) designed as a bihemispheric spiking network, two hemifields receive background oscillations modulated by different frequencies (see Figure 9a). The area encoding relevant information receives oscillations in the theta and gamma bands. This configuration allows the maintenance of the memory (see Figure 9b). During this period, the population encoding irrelevant information receives a background oscillation in the alpha range that prevents a distractor to be encoded (see Figure 9b). This model proposed a potential computational explanation for the lateralization of alpha frequency (see Figure 9c) in delayed-response tasks where relevant and irrelevant information are segregated in different hemifields (Grimault et al., 2009; Sauseng et al., 2009; Van der Werf et al., 2008). Similarly to Dipoppa and Gutkin (2013a), in this version of the model a strong match stimulus directly suppresses the memory trace at the offset of match presentation (see Figure 9b). 

 Interestingly, the limitation of WM capacity has been seen as either a limit on a shared resource or ascribed to existence of discrete slots for distinct items. The framework that we have laid out in this review appears to fall into the discrete slot category by its very construction: two populations for two items. However, extended to cases beyond two items, the model would give behaviour indicative of a common resource model: The probability of task performance, and hence WM precision, would decrease with item number. Interestingly, the contrary behaviour can also been seen—a common resource model can appear to act as a discrete slot one (Wei, Wang, & Wang, 2012). 

 In conclusion, the two oscillation-gating models can perform successfully two different WM tasks with a unified gating mechanism, as opposed to previous models based on unrelated mechanisms regrouped together. Also, the models assign a functional role to a large span of frequencies encompassing the theta, alpha, beta, and gamma bands. We finally stress that, in this paradigm, oscillations have a pure dynamical role, as opposed to other paradigms where oscillations are the carrier of a population code. We note, however, that, at least in a model of signal gating, it has been demonstrated that the two levels are compatible: An information encoded in an oscillatory pattern can be transmitted between two different populations by a mechanism that selects the signals to be gated-in on the basis of their oscillation frequency (Akam & Kullmann, 2010). In our paradigm, the different oscillatory bands ensure that the dynamical state of the WM network is such that all the different operations (or computations) necessary for performing the tasks requiring WM can be implemented by the brain circuits. 

## Discussion

This review has addressed two main issues pertaining to the neural basis of WM. The first is to understand the neural mechanisms that underlie the execution of the required WM operations, namely, load, maintain, prevent, and clear, that enable the execution of a stereotypical delayed response task. To execute these operations, the WM system requires a gating mechanism that selects which information can be encoded into memory or eliminated from it. The second issue is to provide a unifying paradigm that explains the functional role of the different frequency bands that emerge and progress as a sequence in WM. In particular, the goal is to explain the reasons why some bands (theta, beta, and, gamma) are associated with WM maintenance and others with functional inhibition (alpha).

 By proposing different specific mechanisms, previous models based on spiking networks led to an outstanding progress in the understanding of the neural substrate of WM. Several influential models are based on recruitment of inhibitory interneurons that allow memory gating through competition between asynchronous attractor states (Brunel & Wang, 2001; Compte et al., 2000). We defined this paradigm as inhibition-induced gating. While a powerful idea, potentially consistent with local neural circuitry in the PFC, one of the intrinsic drawbacks of inhibition-induced gating is that the related models cannot robustly prevent incorrect memory activation by strong distracting stimuli: For these models to work, distractors would have to evoke less activity than the ongoing memory trace, which is not always the case. Other influential models, based on nested oscillations, have been able to associate theta, beta, and gamma bands with WM maintenance (Kopell et al., 2011; Lisman & Idiart, 1995). Nevertheless, in these models, neither the operations of prevent and clear nor the association of alpha oscillations with functional inhibition are addressed. 

### Oscillation-induced Gating Enables Working Memory Performance

 Dipoppa and Gutkin (2013b) proposed a potential solution to these issues by showing that ongoing oscillations in the WM system can control the gating modes of the WM network in an event-dependent manner, allowing the execution of all the required operations. More specifically, the oscillation frequency modulates the transition probability between the resting state and the persistent state. Hence, since the persistent state is associated with the WMAS while the resting state is associated with the absence of stored memory, these transitions correspond to the WM operations. As we have described in this review, the modulation of the transition probability between the two dynamical states of the WM network (maintenance of active state and ongoing ground state) controls the gating mode of the WMAS. Hence, by varying oscillation frequency, the WM system sets the WMAS network in a particular gating mode where each transition is facilitated or depressed. We have defined this paradigm as oscillation-induced gating. 

The following frequency ranges encompass three complementary gating modes: gate-in mode (memory can be loaded and maintained) within the beta and gamma bands, selective-gate (memory can be maintained but not loaded) within the theta band, and gate-out (memory can neither be loaded nor maintained) within the alpha band. In the model, the WM system switches the frequency during the different stages of the task to set the WMAS in different gating modes.

 The proposed pattern of neural oscillations that emerges during WM processing could explain why theta, beta, and gamma oscillations are involved in memory maintenance (Jensen & Tesche, 2002; Spitzer et al., 2010; Tallon-Baudry et al., 1998), while alpha oscillations are involved in functional inhibition (Jensen et al., 2002; Jokisch & Jensen, 2007). The association of theta, beta, and gamma oscillations with memory maintenance has already been grounded on a neural substrate in the models introduced by Lisman and Idiart (1995) and Kopell et al. (2011) where high-frequency oscillations are nested into low-frequency oscillations to sustain a multiplexed memory. The novelty of the model of Dipoppa and Gutkin (2013b) is that it explains the dichotomy between these three bands and the alpha band. Indeed, the theta band is associated with the selective-gate mode, and the beta and gamma bands are associated with the gate-in mode. In both gating modes, memory is maintained. On the other hand, alpha band is associated with the gate-out mode, where memory cannot be maintained. 

 This work predicts that the probability to block a distractor or erase a memory during WM would be differentially modulated by induced theta versus alpha oscillations. Such oscillations could be induced by rTMSs as in Sauseng et al. ( 2009) or by presenting visual stimuli on a low-contrast grating background oscillating in time at the required frequency. 

### Spatial Modulation of Oscillations in a Bilateral Network

 Dipoppa and Gutkin (2013b) also provide a potential explanation for alpha lateralization that emerges in the neural activity of subjects performing visual bilateral WM tasks (Grimault et al., 2009; Sauseng et al., 2009; Van der Werf et al., 2008). In these tasks, alpha activity increases in the hemisphere ipsilateral to the only hemifield containing the relevant stimuli. Since the ipsilateral hemisphere is thought to be opposed to the hemifield coding for the relevant stimulus (Funahashi, Bruce, et al., 1993), the alpha increase could be related to functional inhibition of irrelevant stimuli. This view is further confirmed by rTMS in the alpha band applied during WM task (Sauseng et al., 2009). To explain such a phenomenon, Dipoppa and Gutkin (2013b) have introduced a distal network where the oscillations are modulated differently in two bilateral areas. The area encoding irrelevant stimuli receives background input modulated by alpha oscillation and is thus set in gate-out mode, preventing incorrect memory activation by distracting stimuli. However, we should treat the comparison of the model with the experimental data with caution. In fact, this hypothesis does not explain alpha lateralization that also emerges in visuospatial attentional bilateral tasks (Haegens et al., 2012; Händel et al., 2011) and where persistent activity may not be involved. 

### Correlation-induced Gating Through Spike Synchronization

 Dipoppa and Gutkin (2013a) show that similarly to the oscillation-induced gating, the gating modes can be obtained by modulating the level of correlation in background activity that in turn modulates the spike-synchronization level (in fact, one can think of coherent oscillations as a spatio-temporal correlation pattern in the ongoing activity). This effect is defined as synchronization-induced gating. The involvement of synchronization in the control of the persistent state stability was previously shown in related work on spatial WM where spike synchronization suppresses the persistent state (Gutkin et al., 2001; Laing & Chow, 2001). In fact, these results suggest that the disruption of the persistent state by a spike-synchronized input is related to the tendency of its spike-time structure to arrange in an asynchronous state. 

 Dipoppa, Krupa, Torcini, and Gutkin (2012) provide a mathematical foundation to this mechanism by studying analytically the spike-time structure of the persistent state. The model consists of a bistable network of excitable (sub-threshold) neurons with all-to-all couplings, so as to allow developing analytical equations. The stable state with the lowest firing rate is the splay state, a highly symmetric state where the spikes of the network are equally spaced in time. Hence, the persistent state with the lowest energy has an asynchronous structure. In contrast, the fully synchronized state is unstable, consistent with the suppression-by-synchronization results (Dipoppa et al., 2012). 

### Unifying the Read-out and Clear Operations

 Dipoppa and Gutkin (2013b, 2013b) propose a mechanism by which the memory operations of read-out and clear may be unified: They have shown that if the match stimulus is strong enough, it deactivates the persistent activity in the corresponding population and thus clears the memory. This effect is not related to the gating mode since it occurs when the system is in the gate-in or selective-gate modes that allow memory maintenance. This is caused by an excess of excitation induced by the match stimulus increasing the level of spike synchronization. Then, similarly to the models of Laing and Chow (2001) and Gutkin et al. (2001), the increase of spike synchronization disrupts the persistent state. Differently from these latter models, the match stimulus is statistically equivalent to the sample stimulus (meaning it activates the same afferents to the WMAS as the sample), and it is not an ad hoc transient increase of excitation with a very short time window. 

 The fact that the match/clear stimulus has the same statistical (e.g., spatial, or the encoding population’s) properties to the sample/encoding stimulus means that the system can react differently to the same stimuli, depending on contextual activity. Since the match stimulus is related to the read-out of information (see Brunel & Wang, 2001 ), this effect unifies the processes of clear and read-out in a single operation. The fact that different operations could be unified is a problem that has been addressed already in other contexts, for example, for parametric WM (Machens et al., 2005). The clear-by-match mechanism could be useful in a task where the network should memorize multiple memories and clear only some of these. In fact, since the match is not global but directed only to the corresponding population, it would not influence the other stored memories. 

### Possible Mechanisms Underlying Oscillation-induced Gating

 One question that arises from the oscillation-induced paradigm is how the multiple frequency switches are obtained in the brain. Oscillating neural activity can shift frequency between different bands under neuromodulatory control, as it has been shown in vitro in slices of the hippocampus (Fellous & Sejnowski, 2000; Whittington, Stanford, Colling, Jeffreys, & Traub , 1997). However, a frequency shift in the same neural population is not strictly required by the oscillation-induced gating paradigm. Another possibility is that different background neural populations that oscillate at distinct frequencies could increase and decrease their activity during the different stages of the task. In this way, at each stage of the task, the dominant oscillation will determine the gating mode of the WM system. 

 One key issue of the oscillation-induced gating paradigm is to determine which area is driving the external oscillations. One possibility is that this role is played by a central executive area. This is in line with the idea that the PFC is a flexible modulator of the WMAS, and it is also compatible with the result that, during a delayed response task, oscillations in distal areas of different frequency bands are synchronized, potentially by a top-down signal (Liebe, Hoerzer, Logothetis, & Rainer, 2012; Sarnthein, Petsche, Rappelsberger, Shaw, & von Stein, 1998). The source of this top-down oscillatory signal could be located in the frontopolar PFC since this area is thought to be involved in control of task contingency (Koechlin et al. , 2003), while the WMAS components could be located in the dorsolateral PFC, in the ITC, or in the parietal cortex, where selective persistent activity has been measured during delayed response tasks (Constantinidis & Steinmetz, 1996; Funahashi et al., 1989; Fuster & Jervey, 1981). Additionally, the lateral prefrontal cortex and its ventro-lateral portions (vLPFC) have been linked to task contingency monitoring and cognitive control (e.g., Koechlin & Hyafil, 2007), making them a likely cortical substrate. 

 The results presented by Dipoppa and Gutkin (2013b) are robust to several parameter variations. The frequency ranges of the gating modes are invariant with respect to changes in the network size, the firing rate of the persistent state, and the duty cycle of the oscillations. The control parameter for all the variables associated with time scales (including frequency bands of the gating modes) is the membrane time constant that has a biologically plausible value of a regular pyramidal neuron, 20 ms in particular for primate PFC (Gonzalez-Burgos, Rotaru, Zaitsev, Povysheva, & Lewis, 2009). The importance of the membrane time constant could be due to its determining of the length of the relative refractory period in our model neuron. 

## Mechanisms of Spike Synchronization During Working Memory Execution

 Modulation of spike synchronization related to WM execution has only recently started to be addressed. For example, it has been shown that when a monkey performs correctly on a DMS task, there is, for most of the delay period, an increase of spike synchrony (Pipa & Munk, 2011). This effect seems consistent with the model of Dipoppa and Gutkin (2013a) where the selective-gate mode, activated during the delay period, requires an increase of correlation level in respect to the gate-in mode, and thus induces an increase of spike synchrony. However, a further experimental analysis should probably be performed on the input correlation to test the predictions of the model. 

 One key issue is to determine the neural substrate of the common background activity source. One possible source is the striatum, a subcortical area thought to be involved in WM. Since the number of striatal neurons is much lower than the number of pyramidal neurons (Lange, Thorner, Hopf, & Schroder, 1976) and the loop is based on divergence (respectively convergence) in the striato-cortical (respectively cortico-striatal) direction, the striatum would not have the same representation capacity as the cortex. Hence, the signals ascending from the striatum would have a lower dimensionality and potentially provide a common input to the relevant PFC WM circuits. It has been suggested that divergent/convergent structure of the striato-cortical loops could be useful, since the basal ganglia do not encode the individual WM representations, but control gating and updates of representations in other brain regions (Frank et al., 2001). It is, therefore, possible that the striatum plays a gating role since it could be the source of the common noise that creates the different regimes. 

### Final Remarks

 In conclusion, in this review, we provided an overview of the various roles for the different oscillatory bands in WM, focusing specifically on the gating and dynamical mechanisms necessary for the network operations inherent in cognitive tasks involving WM. We focused on a set of paradigmatic examples of such tasks (the delay response tasks) and exhibited a novel oscillation- and synchronization-based gating hypothesis. This hypothesis has been put forth in Dipoppa and Gutkin (2013b) who introduced a mechanism combining WM and brain oscillations. The model is able to perform successfully in a WM task with a unified gating mechanism based on oscillations and correlations. Also, the model assigns a dynamical role to a broad span of frequencies, encompassing the theta, alpha, beta, and gamma bands, that synthesize a large number of experimental data. We should stress that in this paradigm oscillations have a pure dynamical role, as opposed to other paradigms where oscillations are the carrier of a population code. Since these two levels of description are not mutually exclusive, it would be interesting to combine them to further understand the function of brain oscillations. This paradigm could open the way to a new approach to the dynamical interaction between oscillations and recurrent networks, and it could lead to the development of new theoretical models and experimental studies. 

## Author Note

Mario Dipoppa was supported by Ministère de l’Enseignement Supérieur et de la Recherche, INSERM, ENS, Wellcome Trust and Marie Curie IEF Grant 627787. Boris S. Gutkin performed this work at the National Research University Higher School of Economics as a result of the project financially supported by the Russian Ministry of Education (Contract no. 14.608.21.0001, unique ID project RFMEFI60815X0001). Marcin Szwed was suported by an NCN grant no.2015/19/B/HS6/01256.

We want to thank Nicolas Brunel, Etienne Koechlin, Catherine Tallon-Baudry, Alexandre Hyafil, David Berrett, Lorenzo Fontolan, Mehdi Keramati, Reinoud Maex, Adrien Wohrer, Matthew Chalk, and Romain Caze.

## References

[R1] Adrian E. D., Matthews B. H. C. (1934). The Berger rhythm potential changes from the occipital lobes in
man.. Brain.

[R2] Akam T., Kullmann D. M. (2010). Oscillations and filtering networks support flexible routing of
information.. Neuron.

[R3] Amit D. J., Brunel N. (1997). Model of global spontaneous activity and local structured
activity during delay periods in the cerebral cortex.. Cerebral Cortex.

[R4] Ashby F. G., Ell S. W., Valentin V. V., Casale M. B. (2005). FROST: A distributed neurocomputational model of working memory
maintenance.. Journal of Cognitive Neuroscience.

[R5a] Baddeley A. (1992). Working memory.. Science.

[R5] Baddeley A., Hitch G. J., Bower G. A., Hitch G. J. (1974). Working memory.. Recent advances in learning and motivation..

[R6] Barrouillet P., Portrat S., Vergauwe E., Diependaele K., Camos V. (2011). Further evidence for temporal decay in working
memory: Reply to Lewandowsky and Oberauer (2009). Journal of Experimental Psychology: Learning, Memory, and Cognition, 37,
1302-1317..

[R6a] Bays P. M., Husain M. (2008). Dynamic shifts of limited working memory resources in human
vision.. Science.

[R7] Berger H. (1929). Über das Elektroenkephalogramm des Menschen [On the
electroencephalogram of man].. Archiv für Psychiatrie und Nervenkrankheiten.

[R8] Bollimunta A., Chen Y., Schroeder C. E., Ding M. (2008). Neuronal mechanisms of cortical alpha oscillations in
awake-behaving macaques.. The Journal of Neuroscience.

[R9] Bollimunta A., Mo J., Schroeder C. E., Ding M. (2011). Neuronal mechanisms and attentional modulation of corticothalamic
alpha oscillations.. The Journal of Neuroscience.

[R10] Brunel N., Wang X. J. (2001). Effects of neuromodulation in a cortical network model of object
working memory dominated by recurrent inhibition.. Journal of Computational Neuroscience.

[R11] Busch N. A., Herrmann C. S. (2003). Object-load and feature-load modulate EEG in a short-term memory
task.. Neuroreport.

[R12] Camperi M., Wang X. J. (1998). A model of visuospatial working memory in prefrontal cortex:
Recurrent network and cellular bistability.. Journal of Computational Neuroscience.

[R12a] Canolty R. T., Edwards E., Dalal S. S., Soltani M., Nagarajan S. S., Kirsch H. E., Knight R. T. (2006). High gamma power is phase-locked to theta oscillations in human
neocortex.. Science.

[R13] Chow S. S., Romo R., Brody C. D. (2009). Context-dependent modulation of functional connectivity:
Secondary somatosensory cortex to prefrontal cortex connections in
two-stimulus-interval discrimination tasks.. The Journal of Neuroscience.

[R14] Cobb W., Muller G. (1954). Parietal focal theta rhythm.. Electroencephalography and Clinical Neurophysiology.

[R15] Cohen J. D., Perlstein W. M., Braver T. S., Nystrom L. E., Noll D. C., Jonides J., Smith E. E. (1997). Temporal dynamics of brain activation during a working memory
task.. Nature.

[R16] Compte A., Brunel N., Goldman-Rakic P. S., Wang X. J. (2000). Synaptic mechanisms and network dynamics underlying spatial
working memory in a cortical network model.. Cerebral Cortex.

[R17] Constantinidis C., Steinmetz M. A. (1996). Neuronal activity in posterior parietal area 7a during the delay
periods of a spatial memory task.. Journal of Neurophysiology.

[R18] Cooper N. R., Burgess A. P., Croft R. J., Gruzelier J. H. (2006). Investigating evoked and induced electroencephalogram activity in
task-related alpha power increases during an internally directed attention
task.. Neuroreport.

[R18a] Courtney S. M., Petit L., Maisog J. M, Ungerleider L. G., Nagarajan S. S., Haxby J. V. (1998). An area specialized for spatial working memory in human frontal
cortex.. Science.

[R19] Courtney S. M., Ungerleider L. G., Keil K., Haxby J. V. (1996). Object and spatial visual working memory activate separate neural
systems in human cortex.. Cerebral Cortex.

[R20] Cowan N. (1995). Attention and memory: An integrated framework..

[R21] Cowan N. (2001). The magical number 4 in short-term memory: A reconsideration of
mental storage capacity.. The Behavioural and Brain Sciences.

[R22] Cowan N. (2008). What are the differences between long-term, short-term, and
working memory?. Progress in Brain Research.

[R23] Daniel R. S. (1967). Alpha and theta EEG in vigilance.. Perceptual and Motor Skills.

[R24] D’Esposito M., Postle B. R. (2015). The cognitive neuroscience of working memory.. Annual Review of Psychology.

[R25] Dipoppa M., Gutkin B. S. (2013a). Correlations in background activity control persistent state
stability and allow execution of working memory tasks.. Frontiers in Computational Neuroscience.

[R26] Dipoppa M., Gutkin B. S. (2013b). Flexible frequency control of cortical oscillations enables
computations required for working memory.. Proceedings of the National Academy of Sciences of the United States of
America.

[R27] Dipoppa M., Krupa M., Torcini A., Gutkin B. S. (2012). Splay states in finite pulse-coupled networks of excitable
neurons.. SIAM Journal on Applied Dynamical Systems.

[R28] Dixon N. F., Lear T. E. (1964). Incidence of theta rhythm prior to awareness of a visual
stimulus.. Nature.

[R29] Fell J., Axmacher N. (2011). The role of phase synchronization in memory
processes.. Nature Reviews Neuroscience.

[R30] Fellous J. M., Sejnowski T. J. (2000). Cholinergic induction of oscillations in the hippocampal slice in
the slow (0.5-2 Hz), theta (5-12 Hz), and gamma (35-70 Hz)
bands.. Hippocampus.

[R31] Frank M. J., Loughry B., O’Reilly R.C. (2001). Interactions between frontal cortex and basal ganglia in working
memory: A computational model.. Cognitive, Affective, and Behavioural Neuroscience.

[R32] Fries P. (2005). A mechanism for cognitive dynamics: Neuronal communication
through neuronal coherence.. Trends in Cognitive Sciences.

[R33] Funahashi S. (2006). Prefrontal cortex and working memory processes.. Neuroscience.

[R34] Funahashi S., Bruce C. J., Goldman-Rakic P. S. (1989).

[R35] Funahashi S., Bruce C. J., Goldman-Rakic P. S. (1993). Dorsolateral prefrontal lesions and oculomotor delayed-response
performance: Evidence for mnemonic “scotomas”.. The Journal of Neuroscience.

[R36] Funahashi S., Chafee M. V., Goldman-Rakic P. S. (1993). Prefrontal neuronal activity in rhesus monkeys performing a
delayed anti-saccade task.. Nature.

[R37] Fuster J. M. (1997). Network memory.. Trends in Neuroscience.

[R37a] Fuster J. M., Alexander G. E. (1971). Neuron activity related to short-term memory.. Science.

[R38] Fuster J. M., Bauer R. H., Jervey J. P. (1981). Effects of cooling inferotemporal cortex on performance of visual
memory tasks.. Experimental Neurology.

[R39] Fuster J. M., Bauer R. H., Jervey J. P. (1982). Cellular discharge in the dorsolateral prefrontal cortex of the
monkey in cognitive tasks.. Experimental Neurology.

[R39a] -Fuster J. M., Jervey J. P. (1981). Inferotemporal neurons distinguish and retain behaviourally
relevant features of visual stimuli.. Science.

[R40] Gevins A., Smith M. E., McEvoy L., Yu D. (1997). High-resolution EEG mapping of cortical activation related to
working memory: Effects of task difficulty, type of processing, and
practice.. Cerebral Cortex.

[R41a] Gevins A. S., Zeitlin G. M., Doyle J. C., Yingling C. D., Schaffer R. E., Callaway R. E., Yeager C. L. (1979). Electroencephalogram correlates of higher cortical
functions.. Science.

[R41] Gevins A. S., Zeitlin G. M., Yingling C. D., Doyle J. C., Dedon M. F., Schaffer R. E., . . . Yeager C. L. (1979). EEG patterns during ‘cognitive’ tasks. I.
Methodology and analysis of complex behaviours.. Electroencephalography and Clinical Neurophysiology.

[R42] Goldman-Rakic P. S. (1995). Cellular basis of working memory.. Neuron.

[R43] Gonzalez-Burgos G., Barrionuevo G., Lewis D. A. (2000). Horizontal synaptic connections in monkey prefrontal cortex: an
in vitro electrophysiological study.. Cerebral Cortex.

[R44] Gonzalez-Burgos G., Rotaru D. C., Zaitsev A. V., Povysheva N. V., Lewis D. A. (2009). GABA transporter GAT1 prevents spillover at proximal and distal
GABA synapses onto primate prefrontal cortex neurons.. Journal of Neurophysiology.

[R45] Gray C. M. (1994). Synchronous oscillations in neuronal systems: Mechanisms and
functions.. Journal of Computational Neuroscience.

[R46] Gray C. M., Singer W. (1989). Stimulus-specific neuronal oscillations in orientation columns of
cat visual-cortex.. Proceedings of the National Academy of Sciences of the United States of
America.

[R47] Grimault S., Robitaille N., Grova C., Lina J. M., Dubarry A. S., Jolicoeur P. (2009). Oscillatory activity in parietal and dorsolateral prefrontal
cortex during retention in visual short-term memory: Additive effects of
spatial attention and memory load.. Human Brain Mapping.

[R48] Gundel A., Wilson G. F. (1992). Topographical changes in the ongoing EEG related to the
difficulty of mental tasks.. Brain Topography.

[R49] Gutkin B. S., Laing C. R., Colby C. L., Chow C. C., Ermentrout G. B. (2001). Turning on and off with excitation: The role of spike-timing
asynchrony and synchrony in sustained neural activity.. Journal of Computational Neuroscience.

[R50] Haegens S., Barczak A., Musacchia G., Lipton M. L., Mehta A. D., Lakatos P., Schroeder C. E. (2015). Laminar profile and physiology of the alpha rhythm in primary
visual, auditory, and somatosensory regions of neocortex.. The Journal of Neuroscience.

[R51] Haegens S., Luther L., Jensen O. (2012). Somatosensory anticipatory alpha activity increases to suppress
distracting input.. Journal of Cognitive Neuroscience.

[R52] Haegens S., Osipova D., Oostenveld R., Jensen O. (2010). Somatosensory working memory performance in humans depends on
both engagement and disengagement of regions in a distributed
network.. Human Brain Mapping.

[R53] Händel B. F., Haarmeier T., Jensen O. (2011). Alpha oscillations correlate with the successful inhibition of
unattended stimuli.. Journal of Cognitive Neuroscience.

[R54] Harris J. A., Miniussi C., Harris I. M., Diamond M. E. (2002). Transient storage of a tactile memory trace in primary
somatosensory cortex.. The Journal of Neuroscience.

[R55] Hasher L., Zacks R. T., May C. P., Gopher D., Koriat A. (1999). Inhibitory control, circadian arousal and age.. Attention and Performance XVII - Cognitive regulation of performance:
Interaction of theory and application.

[R56] Hebb D. O. (1949). The organization of behaviour: A neuropsychological theory..

[R57] Heinrichs-Graham E., Wilson T. W. (2015). Spatiotemporal oscillatory dynamics during the encoding and
maintenance phases of a visual working memory task.. Cortex.

[R58] Hikosaka O., Wurtz R. H. (1983). Visual and oculomotor functions of monkey substantia nigra pars
reticulata. III. Memory-contingent visual and saccade
responses.. Journal of Neurophysiology.

[R59] Hong L. E., Buchanan R. W., Thaker G. K., Shepard P. D., Summerfelt A. (2008). Beta (~16 Hz) frequency neural oscillations mediate auditory
sensory gating in humans.. Psychophysiology.

[R60] Howard M. W., Rizzuto D. S., Caplan J. B., Madsen J. R., Lisman J., Aschenbrenner-Scheibe R., . . . Kahana M. J. (2003). Gamma oscillations correlate with working memory load in
humans.. Cerebral Cortex.

[R61] Hummel F., Gerloff C. (2005). Larger interregional synchrony is associated with greater
behavioural success in a complex sensory integration task in
humans.. Cerebral Cortex.

[R62] Hyafil A., Fontolan L., Kabdebon C., Gutkin B., Giraud A. L. (2015). Speech encoding by coupled cortical theta and gamma oscillations. eLife,
4: e06213..

[R63] Jensen O., Gelfand J., Kounios J., Lisman J. E. (2002). Oscillations in the alpha band (9-12 Hz) increase with memory
load during retention in a short-term memory task.. Cerebral Cortex.

[R64] Jensen O., Idiart M. A., Lisman J. E. (1996). Physiologically realistic formation of autoassociative memory in
networks with theta/gamma oscillations: Role of fast NMDA
channels.. Learning & Memory.

[R65] Jensen O., Lisman J. E. (1996). Novel lists of 7 +/- 2 known items can be reliably stored in an
oscillatory short-term memory network: Interaction with long-term
memory.. Learning & Memory.

[R66] Jensen O., Lisman J. E. (2000). Position reconstruction from an ensemble of hippocampal place
cells: Contribution of theta phase coding.. Journal of Neurophysiology.

[R67] Jensen O., Tesche C. D. (2002). Frontal theta activity in humans increases with memory load in a
working memory task.. The European Journal of Neuroscience.

[R68] Jokisch D., Jensen O. (2007). Modulation of gamma and alpha activity during a working memory
task engaging the dorsal or ventral stream.. The Journal of Neuroscience.

[R69] Kaiser J., Ripper B., Birbaumer N., Lutzenberger W. (2003). Dynamics of gamma-band activity in human magnetoencephalogram
during auditory pattern working memory.. Neuroimage.

[R70] Kilner J. M., Baker S. N., Salenius S., Hari R., Lemon R. N. (2000). Human cortical muscle coherence is directly related to specific
motor parameters.. The Journal of Neuroscience.

[R71] Klimesch W., Doppelmayr M., Schwaiger J., Auinger P., Winkler T. (1999). ‘Paradoxical’ alpha synchronization in a memory
task.. Cognitive Brain Research.

[R72] Klimesch W., Sauseng P., Hanslmayr S. (2007). EEG alpha oscillations: The inhibition-timing
hypothesis.. Brain Research Reviews.

[R72a] Koechlin E., Hyafil A. (2007). Anterior prefrontal function and the limits of human
decision-making.. Science.

[R72b] Koechlin E., Ody C., Kouneiher F. (2003). The architecture of cognitive control in the human prefrontal
cortex.. Science.

[R73] Kopell N., Whittington M. A., Kramer M. A. (2011). Neuronal assembly dynamics in the beta1 frequency range permits
short-term memory.. Proceedings of the National Academy of Sciences of the United States of
America.

[R74] Kremkow J., Aertsen A., Kumar A. (2010). Gating of signal propagation in spiking neural networks by
balanced and correlated excitation and inhibition.. The Journal of Neuroscience.

[R75] Kubota K., Niki H. (1971). Prefrontal cortical unit activity and delayed alternation
performance in monkeys.. Journal of Neurophysiology.

[R76] Kuo B. C., Rao A., Lepsien J., Nobre A. C. (2009). Searching for targets within the spatial layout of visual
short-term memory.. The Journal of Neuroscience.

[R77] Laing C. R., Chow C. C. (2001). Stationary bumps in networks of spiking neurons.. Neural Computation.

[R77a] Landfield P. W., McGaugh J. L., Tusa R. J. (1972). Theta rhythm: A temporal correlate of memory storage processes in
the rat.. Science.

[R78] Lange H., Thorner G., Hopf A., Schroder K. F. (1976). Morphometric studies of the neuropathological changes in
choreatic diseases.. Journal of the Neurological Sciences.

[R79] Lee H., Simpson G. V., Logothetis N. K., Rainer G. (2005). Phase locking of single neuron activity to theta oscillations
during working memory in monkey extrastriate visual cortex.. Neuron.

[R80] Lee S. H., Kravitz D. J., Baker C. I. (2013). Goal-dependent dissociation of visual and prefrontal cortices
during working memory.. Nature Neuroscience.

[R81] Liebe S., Hoerzer G. M., Logothetis N. K., Rainer G. (2012). Theta coupling between V4 and prefrontal cortex predicts visual
short-term memory performance.. Nature Neuroscience.

[R81a] Lisman J. E., Idiart M. A. (1995). Storage of 7 +/- 2 short-term memories in oscillatory
subcycles.. Science.

[R82] Luck S. J., Vogel E. K. (1997). The capacity of visual working memory for features and
conjunctions.. Nature.

[R83] Lutzenberger W., Ripper B., Busse L., Birbaumer N., Kaiser J. (2002). Dynamics of gamma-band activity during an audiospatial working
memory task in humans.. The Journal of Neuroscience.

[R84] Ma W. J., Husain M., Bays P. M. (2014). Changing concepts of working memory.. Nature Neuroscience.

[R84a] Machens C. K., Romo R., Brody C. D. (2005). Flexible control of mutual inhibition: A neural model of
two-interval discrimination.. Science.

[R85] Makeig S., Jung T. P. (1996). Tonic, phasic, and transient EEG correlates of auditory awareness
in drowsiness.. Cognitive Brain Research.

[R86] Melcher D., Piazza M. (2011). The role of attentional priority and saliency in determining
capacity limits in enumeration and visual working memory.. PloS One.

[R87a] Miller E. K., Desimone R. (1994). Parallel neuronal mechanisms for short-term
memory.. Science.

[R87b] Miller E. K., Li L., Desimone R. (1991). A neural mechanism for working and recognition memory in inferior
temporal cortex.. Science.

[R87] Miller E. K., Erickson C .A., Desimone R. (1996). Neural mechanisms of visual working memory in prefrontal cortex
of the macaque.. The Journal of Neuroscience.

[R88a] Miller G. A., Desimone R. (1991). A neural mechanism for working and recognition memory in inferior
temporal cortex.. Science.

[R88] Miller G. A. (1956). The magical number seven plus or minus two: Some limits on our
capacity for processing information.. Psychological Review.

[R89] Mo J., Schroeder C. E., Ding M. (2011). Attentional modulation of alpha oscillations in macaque
inferotemporal cortex.. The Journal of Neuroscience.

[R89a] Mongillo G., Barak O., Tsodyks M. (2008). Synaptic theory of working memory.. Science.

[R90] Murthy V. N., Fetz E. E. (1992). Coherent 25- to 35-Hz oscillations in the sensorimotor cortex of
awake behaving monkeys.. Proceedings of the National Academy of Sciences of the United States of
America.

[R91] Nobre A. C., Coull J. T., Maquet P., Frith C. D., Vandenberghe R., Mesulam M. M. (2004). Orienting attention to locations in perceptual versus mental
representations.. Journal of Cognitive Neuroscience.

[R92] Oberauer K. (2001). Removing irrelevant information from working memory: A cognitive
aging study with the modified Sternberg task.. Journal of Experimental Psychology: Learning, Memory, and
Cognition.

[R93] Oberauer K., Lewandowsky S. (2014). Further evidence against decay in working memory.. Journal of Memory and Language.

[R94] O’Keefe J., Recce M. L. (1993). Phase relationship between hippocampal place units and the EEG
theta rhythm.. Hippocampus.

[R95] Oliveri M., Turriziani P., Carlesimo G. A., Koch G., Tomaiuolo F., Panella M., Caltagirone C. (2001). Parieto-frontal interactions in visual-object and visual-spatial
working memory: Evidence from transcranial magnetic
stimulation.. Cerebral Cortex.

[R96] Osipova D., Hermes D., Jensen O. (2008). Gamma power is phase-locked to posterior alpha
activity.. PloS One.

[R97] Palva S., Linkenkaer-Hansen K., Näätänen R., Palva J. M. (2005). Early neural correlates of conscious somatosensory
perception.. The Journal of Neuroscience.

[R98] Palva S., Palva J. M. (2007). New vistas for alpha-frequency band oscillations.. Trends in Neuroscience.

[R99] Pesaran B., Pezaris J. S., Sahani M., Mitra P. P., Andersen R. A. (2002). Temporal structure in neuronal activity during working memory in
macaque parietal cortex.. Nature Neuroscience.

[R100] Pipa G., Munk M. H. (2011). Higher order spike synchrony in prefrontal cortex during visual
memory.. Frontiers in Computational Neuroscience.

[R101] Pipa G., Stadtler E. S., Rodriguez E. F., Waltz J. A., Muckli L. F., Singer W., . . . Munk M. H. (2009). Performance- and stimulus-dependent oscillations in monkey
prefrontal cortex during short-term memory.. Frontiers in Integrative Neuroscience.

[R102] Polania R., Paulus W., Nitsche M. A. (2012). Noninvasively decoding the contents of visual working memory in
the human prefrontal cortex within high-gamma oscillatory
patterns.. Journal of Cognitive Neuroscience.

[R103] Raghavachari S., Kahana M. J., Rizzuto D. S., Caplan J. B., Kirschen M. P., Bourgeois B., . . . Lisman J. E. (2001). Gating of human theta oscillations by a working memory
task.. The Journal of Neuroscience.

[R104] Riggall A. C., Postle B. R. (2012). The relationship between working memory storage and elevated
activity as measured with functional magnetic resonance
imaging.. The Journal of Neuroscience.

[R105] Rigotti M., Barak O., Warden M. R., Wang X. J., Daw N. D., Miller E. K., Fusi S. (2013). The importance of mixed selectivity in complex cognitive
tasks.. Nature.

[R106] Riley M. R., Constantinidis C. (2015). Role of prefrontal persistent activity in working
memory.. Frontiers in Systems Neuroscience.

[R107] Romo R., Brody C. D., Hernandez A., Lemus L. (1999). Neuronal correlates of parametric working memory in the
prefrontal cortex.. Nature.

[R108] Roux F., Uhlhaas P. J. (2014). Working memory and neural oscillations: Alpha-gamma versus
theta-gamma codes for distinct WM information?. Trends in Cognitive Sciences.

[R109] Roux F., Wibral M., Mohr H. M., Singer W., Uhlhaas P. J. (2012). Gamma-band activity in human prefrontal cortex codes for the
number of relevant items maintained in working memory.. The Journal of Neuroscience.

[R110] Sakurai Y., Takahashi S. (2006). Dynamic synchrony of firing in the monkey prefrontal cortex
during working-memory tasks.. The Journal of Neuroscience.

[R111] Sarnthein J., Petsche H., Rappelsberger P., Shaw G. L., von Stein A. (1998). Synchronization between prefrontal and posterior association
cortex during human working memory.. Proceedings of the National Academy of Sciences of the United States of
America.

[R112] Sauseng P., Klimesch W., Doppelmayr M., Pecherstorfer T., Freunberger R., Hanslmayr S. (2005). EEG alpha synchronization and functional coupling during top-down
processing in a working memory task.. Human Brain Mapping.

[R113] Sauseng P., Klimesch W., Heise K. F., Gruber W. R., Holz E., Karim A. A., . . . Hummel F. C. (2009). Brain oscillatory substrates of visual short-term memory
capacity.. Current Biology.

[R114] Serences J. T., Ester E. F., Vogel E. K., Awh E. (2009). Stimulus-specific delay activity in human primary visual
cortex.. Psychological Science.

[R115] Shao N., Li J., Shui R., Zheng X., Lu J., Shen M. (2010). Saccades elicit obligatory allocation of visual working
memory.. Memory & Cognition.

[R116] Spitzer B., Wacker E., Blankenburg F. (2010). Oscillatory correlates of vibrotactile frequency processing in
human working memory.. The Journal of Neuroscience.

[R116a] Sternberg S. (1966). High-speed scanning in human memory.. Science.

[R117] Stokes M. G. (2015). ‘Activity-silent’ working memory in prefrontal
cortex: A dynamic coding framework.. Trends in Cognitive Sciences.

[R118] Szatmary B., Izhikevich E. M. (2010). Spike-timing theory of working memory.. PLoS Computational Biology.

[R119] Tallon-Baudry C., Bertrand O., Peronnet F., Pernier J. (1998). Induced gamma-band activity during the delay of a visual
short-term memory task in humans.. The Journal of Neuroscience.

[R120] Tallon-Baudry C., Kreiter A., Bertrand O. (1999). Sustained and transient oscillatory responses in the gamma and
beta bands in a visual short-term memory task in humans.. Visual Neuroscience.

[R121] Tallon-Baudry C., Mandon S., Freiwald W. A., Kreiter A. K. (2004). Oscillatory synchrony in the monkey temporal lobe correlates with
performance in a visual short-term memory task.. Cerebral Cortex.

[R122] Tesche C. D., Karhu J. (2000). Theta oscillations index human hippocampal activation during a
working memory task.. Proceedings of the National Academy of Sciences of the United States of
America.

[R123] Uhlhaas P. J., Haenschel C., Nikolic D., Singer W. (2008). The role of oscillations and synchrony in cortical networks and
their putative relevance for the pathophysiology of
schizophrenia.. Schizophrenia Bulletin.

[R124] Van den Bergh G., Zhang B., Arckens L., Chino Y. M. (2010). Receptive-field properties of V1 and V2 neurons in mice and
macaque monkeys.. The Journal of Comparative Neurology.

[R125] Van der Lubbe R. H., Bundt C., Abrahamse E. L. (2014). Internal and external spatial attention examined with lateralized
EEG power spectra.. Brain Research.

[R126] Van der Lubbe R. H., Utzerath C. (2013). Lateralized power spectra of the EEG as an index of visuospatial
attention.. Advances in Cognitive Psychology.

[R127] Van der Werf J., Jensen O., Fries P., Medendorp W. P. (2008). Gamma-band activity in human posterior parietal cortex encodes
the motor goal during delayed prosaccades and antisaccades.. Journal of Neuroscience.

[R128] VanRullen R., Koch C. (2003). Is perception discrete or continuous?. Trends in Cognitive Sciences.

[R129] Van Vreeswijk C., Abbott L. F., Ermentrout G. B. (1994). When inhibition not excitation synchronizes neural
firing.. Journal of Computational Neuroscience.

[R130] Varela F. J., Toro A., John E. R., Schwartz E. L. (1981). Perceptual framing and cortical alpha rhythm.. Neuropsychologia.

[R131] Vogels T. P., Abbott L. F. (2009). Gating multiple signals through detailed balance of excitation
and inhibition in spiking networks.. Nature Neuroscience.

[R132] Voytek B., Canolty R. T., Shestyuk A., Crone N. E., Parvizi J., Knight R. T. (2010). Shifts in gamma phase-amplitude coupling frequency from theta to
alpha over posterior cortex during visual tasks.. Frontiers in Human Neuroscience.

[R133] Wang X. J. (2010). Neurophysiological and computational principles of cortical
rhythms in cognition.. Physiological Review.

[R134] Warden M. R., Miller E. K. (2010). Task-dependent changes in short-term memory in the prefrontal
cortex.. The Journal of Neuroscience.

[R135] Wei Z., Wang X. J., Wang D. H. (2012). From distributed resources to limited slots in multiple-item
working memory: A spiking network model with normalization.. The Journal of Neuroscience.

[R136] Whittington M. A., Stanford I. M., Colling S. B., Jefferys J. G., Traub R. D. (1997). Spatiotemporal patterns of gamma frequency oscillations
tetanically induced in the rat hippocampal slice.. The Journal of Physiology.

[R137] Wilken P., Ma W. J. (2004). A detection theory account of change detection.. Journal of Vision.

[R138] Wimmer K., Nykamp D. Q., Constantinidis C., Compte A. (2014). Bump attractor dynamics in prefrontal cortex explains behavioural
precision in spatial working memory.. Nature Neuroscience.

[R139] Wimmer K., Ramon M., Pasternak T., Compte A. (2016). Transitions between multiband oscillatory patterns characterize
memory-guided perceptual decisions in prefrontal circuits.. Journal of Neuroscience.

[R140] Womelsdorf T., Valiante T. A., Sahin N. T., Miller K. J., Tiesinga P. (2014). Dynamic circuit motifs underlying rhythmic gain control, gating,
and integration.. Nature Neuroscience.

[R141] Worden M. S., Foxe J. J., Wang N., Simpson G. V. (2000). Anticipatory biasing of visuospatial attention indexed by
retinotopically specific alpha-band electroencephalography increases over
occipital cortex.. The Journal of Neuroscience.

[R142] Wu W., Wheeler D. W., Staedtler E. S., Munk M. H., Pipa G. (2008). Behavioural performance modulates spike field coherence in monkey
prefrontal cortex.. Neuroreport.

[R143] Zhang W., Luck S. J. (2008). Discrete fixed-resolution representations in visual working
memory.. Nature.

[R144] Zhou Y. D., Fuster J. M. (1996). Mnemonic neuronal activity in somatosensory
cortex.. Proceedings of the National Academy of Sciences of the United States of
America.

